# SIRT6 protects vascular smooth muscle cells from osteogenic transdifferentiation via Runx2 in chronic kidney disease

**DOI:** 10.1172/JCI150051

**Published:** 2022-01-04

**Authors:** Wenxin Li, Weijing Feng, Xiaoyan Su, Dongling Luo, Zhibing Li, Yongqiao Zhou, Yongjun Zhu, Mengbi Zhang, Jie Chen, Baohua Liu, Hui Huang

**Affiliations:** 1 Department of Cardiology, The Eighth Affiliated Hospital, Sun Yat-sen University, Shenzhen, China.; 2 Department of Cardiology, State Key Laboratory of Organ Failure Research, Guangdong Provincial Key Lab of Shock and Microcirculation, Nanfang Hospital, Southern Medical University, Guangzhou, China.; 3 Nephropathy Department, Tungwah Hospital of Sun Yat-sen University, Dongguan, China.; 4 Department of Cardiology, Sun Yat-sen Memorial Hospital, Sun Yat-sen University, Guangzhou, China.; 5 Department of Radiation Oncology, Sun Yat-sen Memorial Hospital, Sun Yat-sen University, Guangzhou, China.; 6 Shenzhen Key Laboratory for Systemic Aging and Intervention, National Engineering Research Center for Biotechnology-Shenzhen, Shenzhen University Health Science Center, Shenzhen, China.

**Keywords:** Cell Biology, Vascular Biology, Cardiovascular disease, Chronic kidney disease, Ubiquitin-proteosome system

## Abstract

Vascular calcification (VC) is regarded as an important pathological change lacking effective treatment and associated with high mortality. Sirtuin 6 (SIRT6) is a member of the Sirtuin family, a class III histone deacetylase and a key epigenetic regulator. SIRT6 has a protective role in patients with chronic kidney disease (CKD). However, the exact role and molecular mechanism of SIRT6 in VC in patients with CKD remain unclear. Here, we demonstrated that SIRT6 was markedly downregulated in peripheral blood mononuclear cells (PBMCs) and in the radial artery tissue of patients with CKD with VC. SIRT6-transgenic (SIRT6-Tg) mice showed alleviated VC, while vascular smooth muscle cell–specific (VSMC-specific) SIRT6 knocked-down mice showed severe VC in CKD. SIRT6 suppressed the osteogenic transdifferentiation of VSMCs via regulation of runt-related transcription factor 2 (Runx2). Coimmunoprecipitation (co-IP) and immunoprecipitation (IP) assays confirmed that SIRT6 bound to Runx2. Moreover, Runx2 was deacetylated by SIRT6 and further promoted nuclear export via exportin 1 (XPO1), which in turn caused degradation of Runx2 through the ubiquitin-proteasome system. These results demonstrated that SIRT6 prevented VC by suppressing the osteogenic transdifferentiation of VSMCs, and as such targeting SIRT6 may be an appealing therapeutic target for VC in CKD.

## Graphical abstract

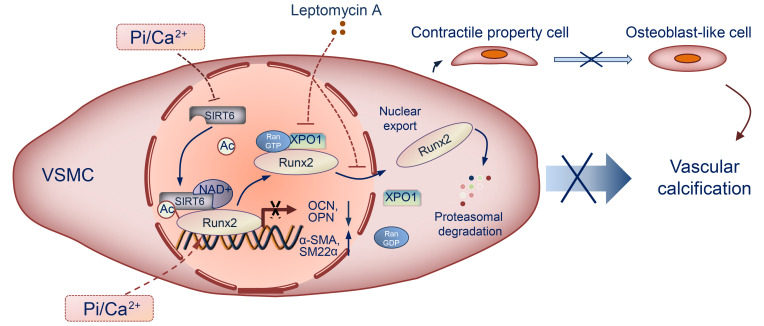

## Introduction

Vascular calcification (VC), especially in tunica media, is prevalent in patients with chronic kidney disease (CKD) (1–3). Previous research has revealed that VC is a major contributor to major adverse cardiovascular events in CKD and thus is considered an important pathological change in cardiovascular disease (4–6). Despite severe clinical consequences, the molecular mechanism underlying VC remains ill defined and no effective therapeutic strategies are currently available to prevent or halt the progression of VC in CKD. Recent studies suggest that VC in CKD is a complex and highly regulated process. Patients with CKD develop hyperphosphatemia, which subsequently promotes the osteogenic transdifferentiation of vascular smooth muscle cells (VSMCs) (5, 7, 8). Phosphate-induced (Pi-induced) remodeling of VSMCs is essential for the mineralization of vascular tissue, and is highly regulated by osteogenic transcription factor runt-related transcription factor 2 (Runx2, also known as core-binding factor subunit α1, CBFA1; refs. 9–12). In this context, it is tempting to suggest that treatment strategies are needed to block osteogenic transdifferentiation of VSMCs for preventing or halting the progression of VC. However, inhibition of osteogenic transdifferentiation of VSMCs has not been developed, and such approaches are still lacking.

Sirtuins (SIRTs) are conserved NAD^+^-dependent protein deacetylases that have beneficial effects against aging and metabolic diseases, and have been recognized as a potential effective target for cardiovascular disease (CVD) (13–17). They can maintain endothelial homeostatic functions, delay vascular aging (18), and protect cardiomyocyte against cardiomyocyte hypertrophy (19). In addition, SIRTs also show a protective role in CKD (20–22). This moderating effect may indicate that SIRTs are involved in CVD associated with CKD. Therefore, further understanding of the functional mechanism of SIRTs to serve as a therapeutic target for CVD, especially in CKD, is needed.

This study explored the role and underlying molecular mechanism of SIRT6 in VC induced by CKD. Using clinical samples from patients with CKD, we identified that SIRT6 was decreased in PBMCs and calcified arteries. We explored the effect of SIRT6 on VC in CKD and osteogenic transdifferentiation of VSMCs both in vivo and in vitro. We verified that SIRT6 prevented VC in our models, and elaborated on the molecular mechanism by which it does so. These findings highlight the critical role of SIRT6 in VC and indicate that SIRT6 may act as a novel potential therapeutic target for VC in CKD.

## Results

### SIRT6 reduction is associated with increased risk of VC in patients with CKD.

**Figure 1:**
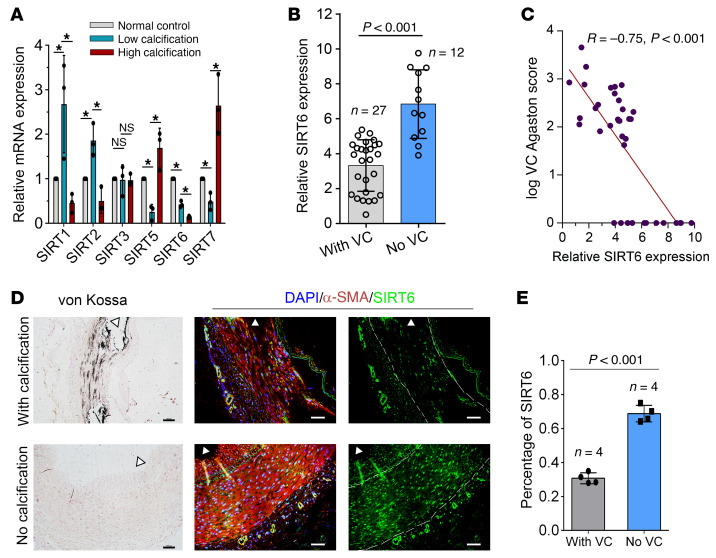
Low level of SIRT6 expression was associated with increased risk of vascular calcification. (**A**) The qPCR showed expression of SIRT1-7 in WT VSMCs with different calcification statuses. SIRT4 was not detected in VSMCs (*n =* 4 per group). Data were expressed as mean ± SD, *P < 0.05. (**B**) SIRT6 mRNA levels in PBMCs from patients with CKD with (*n =* 27) or without (*n =* 12) VC. Data were expressed as mean ± SD. (**C**) Correlation between the SIRT6 mRNA level and VC scores in patients with CKD (*n =* 39, the Pearson’s correlation coefficient *R* value and the *P* value are shown). (**D**) von Kossa assay and IF staining for SIRT6 in radial arteries sections from hemodialysis patients with CKD (*n =* 4 per group). Scale bars: von Kossa 100 μm; IF 50 μm. (**E**) The bars showing SIRT6 protein expression (mean ± SD; *n =* 4 per group; AU) in nuclei of aortic tissues between patients with CKD with and without VC. Statistical significance was assessed using 1-way ANOVA followed by Dunnett’s test (**A**) and 2-tailed *t* tests (**B** and **E**).

The expression levels of the sirtuins family (SIRT1-7) in primary human aortic smooth muscle cells (HAoSMCs) with different calcification status induced by Pi were detected (Supplemental Figure 1A; supplemental material available online with this article; https://doi.org/10.1172/JCI150051DS1). As shown in Figure 1A, the mRNA expression of SIRT6 was the only significantly downregulated SIRT at different calcification levels. To investigate the association between SIRT6 and VC, SIRT6 expression was detected by using the mRNA of PBMCs in 39 patients with CKD with or without VC and 20 healthy people. Patients with CKD presented lower SIRT6 expression compared with healthy people (Supplemental Figure 1B). Patients with VC had significantly lower levels of SIRT6 (3.32 ± 1.47 vs. 6.84 ± 1.96, *P* < 0.001; Figure 1B) and higher body mass index (24.94 ± 4.06 vs. 22.02 ± 2.10, *P* = 0.02; Table 1) than those without VC. SIRT6 expression was inversely correlated with VC Agatston scores of thoracic aorta (*P* < 0.001; Figure 1C). There were no differences in age, sex distribution proportion, kidney function, and traditional risk factors between the groups with and without VC (systolic blood pressure [SBP], diastolic blood pressure [DBP], and lipid profile) (Table 1). Von Kossa assays were performed to verify VC, in addition to immunofluorescence (IF) staining for SIRT6 in radial arteries from patients undergoing hemodialysis. In tunica media, SIRT6 expression was detected in more than 65% of nuclei with no calcification of the arteries, while it exhibited significantly lower expression (about 30% nuclei) in calcified arteries (Figure 1, D and E). These data indicated that SIRT6 expression decreased in VC among patients with CKD.

### SIRT6 impedes vascular calcification in vivo and in vitro.

**Figure 2:**
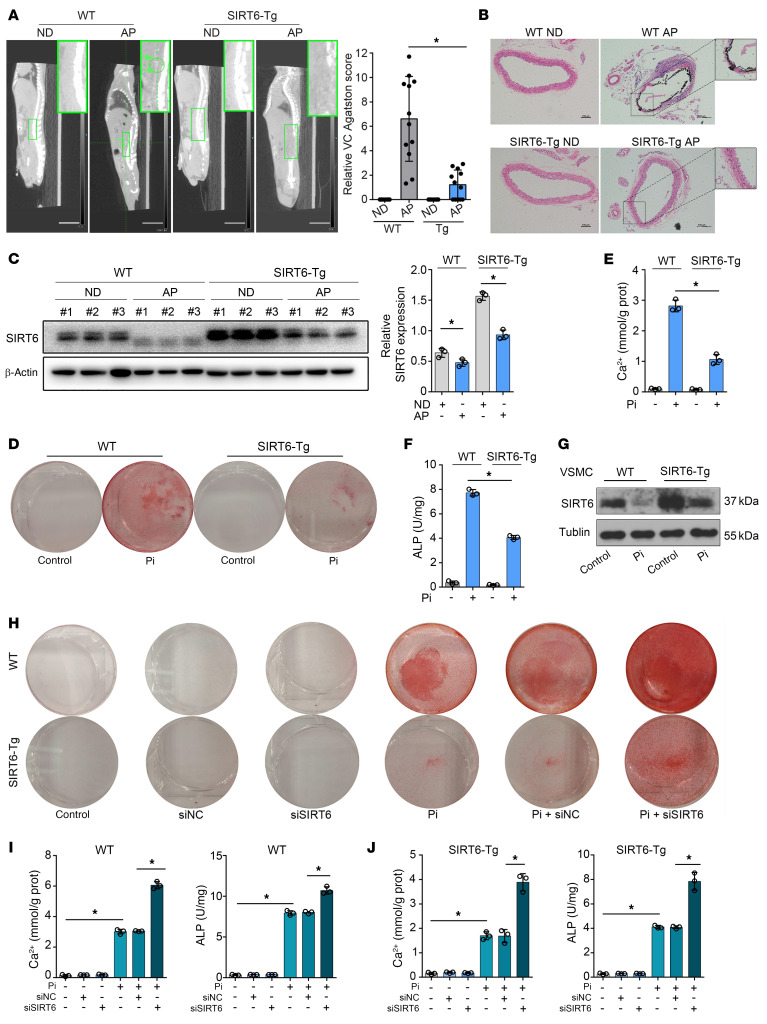
SIRT6 attenuated VC. (**A**) Computed tomography (CT) images showing calcification in the abdominal aorta. The green arrows and circle indicated the calcification in abdominal aorta of the WT mouse (*n =* 12 per group). The bar chart shows the relative VC Agatston score (fold change) of mouse aortas. Scale bars: 10 mm. (**B**) Representative von Kossa staining of abdominal aorta sections (*n =* 12 per group). Scale bars: 100 μm. (**C**) Western blot shows SIRT6 protein in abdominal aorta was reduced in VC. (**D **and** E**) VSMCs were exposed to Pi (3.0 mM) for 7 days and then stained for mineralization by Alizarin red (**D**), and the quantitative analysis of calcium content (**E**) and ALP (**F**) were detected (*n =* 3 per group). (**G**) SIRT6 protein expression was reduced in WT and SIRT6-Tg VSMCs in response to Pi (3.0 mM) treatment (*n =* 4 per group). (**H**–**J**) WT and SIRT6-Tg VSMCs were pretransfected with siSIRT6 or si-negative control (siNC) and then exposed to Pi (3.0 mM) for 7 days. VSMCs were stained for mineralization by Alizarin red S (**H**), and calcium content (**I**) and ALP (**J**) were quantified (*n =* 3 per group). Statistical significance was assessed using 1-way ANOVA followed by Dunnett’s test (**A**,** C**–**F**,** I**, and **J**). *P < 0.05. All values are mean ± SD.

To gain insight of the role of SIRT6 in VC, we induced VC through 2 CKD models (adenine and phosphorus diet–induced [AP-induced] mode and 5/6 nephrectomy mode) in WT mice. CKD status promoted VC in WT mice (Figure 2, A and B; Supplemental Figure 2, A–C). SIRT6 protein expression in calcified aortas was decreased compared with healthy controls (Figure 2C and Supplemental Figure 2D). We then generated the SIRT6-transgenic mice (SIRT6-Tg, for stable overexpression of SIRT6) and subsequently induced VC through CKD status. SIRT6 expression was enhanced in the aorta of SIRT6-Tg mice (Supplemental Figure 3). Calcification in aorta was reduced significantly in SIRT6-Tg mice (Figure 2, A and B and Supplemental Figure 2A). Of note, SIRT6 protein expression in calcified aortas was also decreased in SIRT6-Tg mice, similar to WT mice (Figure 2C and Supplemental Figure 2E). Furthermore, we used adeno-associated viral (serotype 2 gene, AAV2) to specifically knock down SIRT6 expression in VSMCs. AAV2-sh-SIRT6 successfully reduced SIRT6 expression in aorta but there was no change in kidney (Supplemental Figure 4). As expected, SIRT6 reduction in aorta induced severe VC in CKD status (Supplemental Figure 5, A–C).

To better understand the role of SIRT6 in regulating VC, we constructed experiments on primary VSMCs in vitro. The VSMCs were identified by smooth muscle myosin heavy chain and SM22α (Supplemental Figure 6). Treated with Pi (3.0 mmol/L), SIRT6-Tg VSMCs exhibited lower calcium deposition than WT VSMCs, as evidenced by Alizarin red staining, calcium content assay, and alkaline phosphatase (ALP) (Figure 2, D–F). SIRT6 expression decreased upon VSMC calcification (Figure 2G). Furthermore, in vitro loss-of-function analyses were performed using small interfering RNA (siRNA) or specific SIRT6 inhibitor OSS-128167. SIRT6 expression was successfully suppressed (Supplemental Figure 7, A and B). Silencing of SIRT6 in VSMCs resulted in severe calcium deposition and increased ALP (Figure 2, H–J and Supplemental Figure 7, C–E), which indicated that SIRT6 deficiency aggravated VC. Collectively, these data suggested that SIRT6 played a protective role against VC in vivo and in vitro.

### SIRT6 suppresses osteogenic transdifferentiation of VSMCs via downregulation of Runx2.

**Figure 3:**
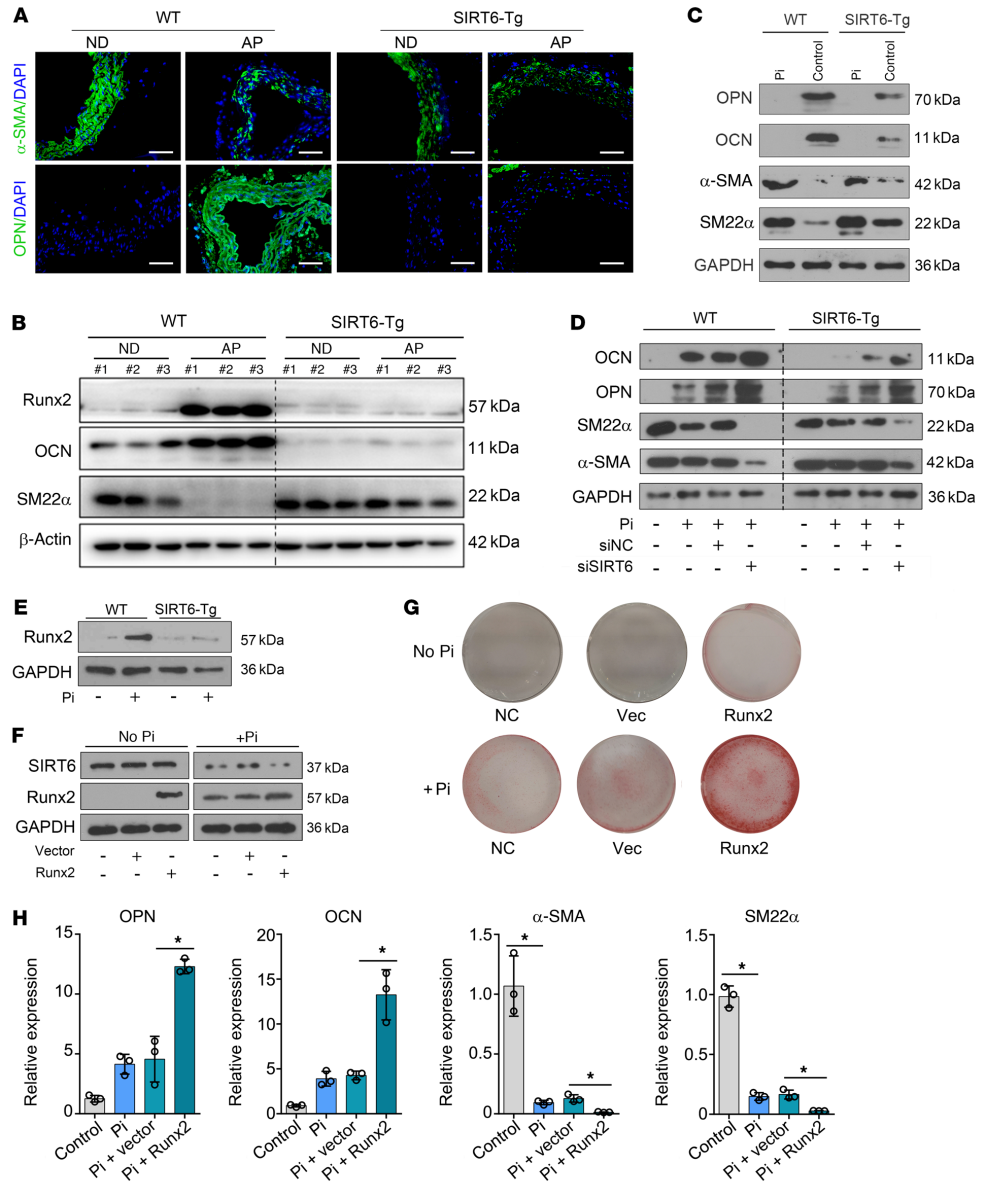
SIRT6 suppresses osteogenic transdifferentiation of VSMCs via regulation of Runx2. (**A**) Expression levels of α-SMA and OPN in abdominal arteries of indicated groups were determined by IF staining (*n =* 4 per group). Scale bars: 50 μm. (**B**) Western blot analysis of osteogenic and contractile property factors expression in abdominal arteries (*n =* 3 per group). (**C**) Analysis of osteogenic and contractile property factor expression in WT and SIRT6-Tg VSMCs after Pi (3.0 mM) treatment by Western blot (*n =* 4 per group). (**D**) VSMCs were pretransfected with siSIRT6 or siNC, and then incubated with Pi (3.0 mM) for 7 days, and the downstream osteogenic markers (OPN, OCN) and contractile property markers (α-SMA, SM22α) were analyzed by Western blot (*n =* 4 per group). (**E**) Runx2 expression was analyzed in WT and SIRT6-Tg VSMCs after Pi (3.0 mM) treatment by Western blot (*n =* 4 per group). (**F**–**H**) SIRT6-Tg VSMCs were pretransfected with Runx2 plasmid or vector plasmid, and then exposed to Pi (3.0 mM) for 7 days. The expression of SIRT6 and Runx2 were analyzed by Western blot (**F**). VSMCs were stained for mineralization by Alizarin red S (**G**), and osteogenic markers (OPN, OCN) and contractile property markers (α-SMA, SM22α) were analyzed by qPCR (*n =* 3 per group) (**H**). Statistical significance was assessed using 1-way ANOVA followed by Dunnett’s test (**H**). *P < 0.05. All values are mean ± SD.

Osteogenic transdifferentiation of VSMCs serve a critical role in VC, so we explored the potential role of SIRT6 in this process. SIRT6 reduced the expression of osteogenic markers osteopontin (OPN) and osteocalcin (OCN) and maintained the expression of contractile property markers α-smooth muscle actin (α-SMA) and smooth muscle-22α (SM22α) in vivo (Figure 3, A and B and Supplemental Figure 2F). As expected, SIRT6 restrained the reduction of SM22α and α-SMA, and downregulated OPN and OCN in SIRT6-Tg VSMCs when treated with Pi in vitro (Figure 3C and Supplemental Figure 8A). Conversely, the contractile markers decreased while osteogenic markers increased in VSMCs when treated with siSIRT6 and OSS-128167 (Figure 3D; Supplemental Figure 8, B–E and Supplemental Figure 9A). The same results were observed in AAV2-treated mice. SIRT6 deficiency promoted osteogenic transdifferentiation of VSMCs in CKD mice (Supplemental Figure 5, D and E). Taken together, these results suggested that SIRT6 protected against VC by suppressing osteogenic transdifferentiation of VSMCs.

Since the osteogenic transdifferentiation of VSMCs was highly regulated by Runx2 (9, 10), we next examined whether SIRT6 regulated VC through Runx2. Runx2 expression was much lower in the SIRT6-Tg group in vivo and in vitro (Figure 3, B and E and Supplemental Figure 2F). Interestingly, the mRNA expression level of Runx2 had no marked change between the 2 groups (Supplemental Figure 9D). Additionally, overexpression of Runx2 removed the protective capacity of SIRT6 (Figure 3, F–H and Supplemental Figure 9, B and C). These results demonstrated that SIRT6 suppressed osteogenic transdifferentiation of VSMCs via downregulation of Runx2.

### SIRT6 deacetylates Runx2 in osteogenic transdifferentiation of VSMCs.

**Figure 4:**
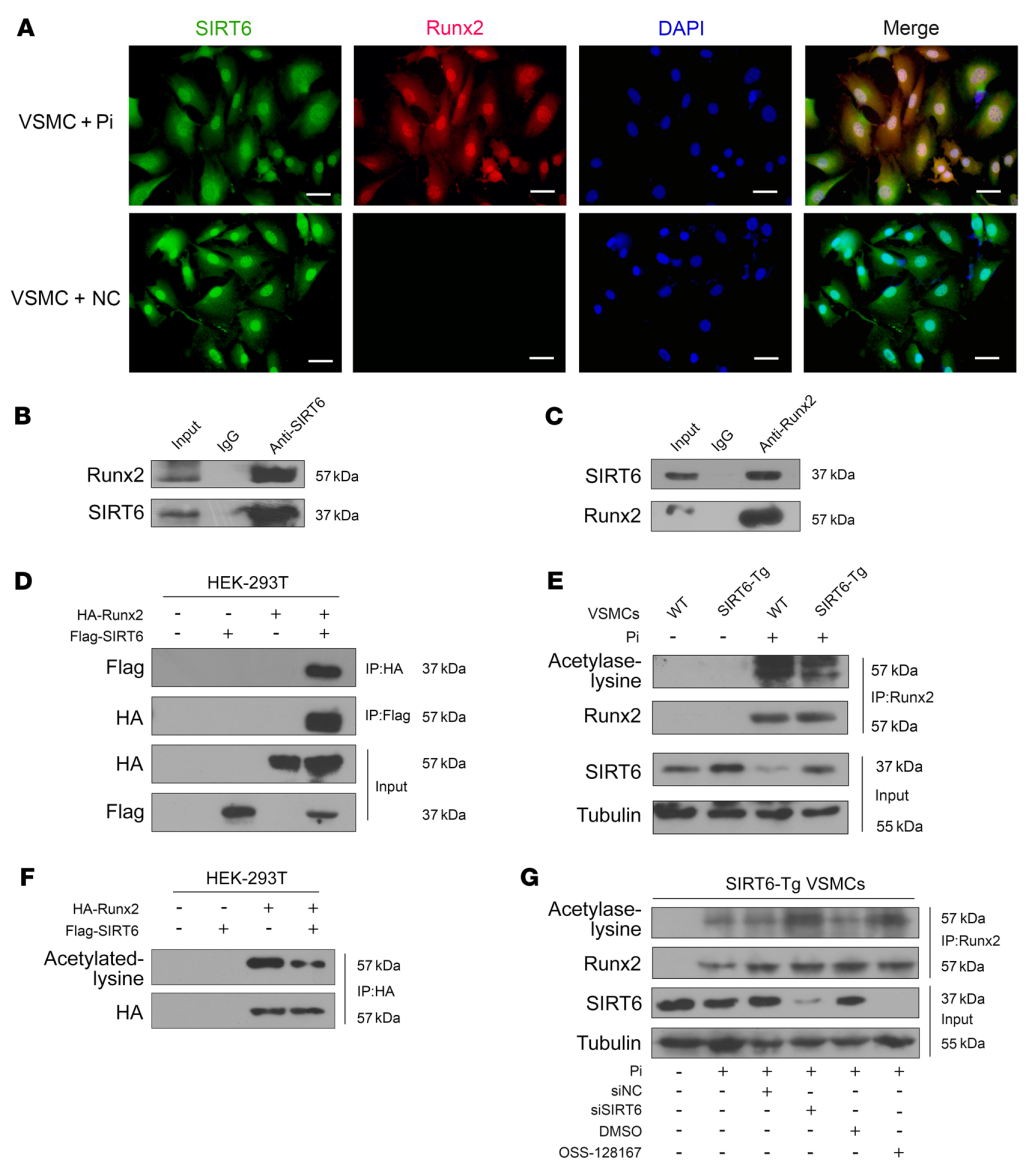
SIRT6 deacetylates Runx2. (**A**) Representative IF images showing the colocalization of SIRT6 and Runx2. Scale bars: 50 μm. (**B**) Anti-SIRT6 IP followed by Western blot with anti-Runx2 or anti-SIRT6 antibody in SIRT6-Tg VSMCs after treatment with Pi (3.0 mM) for 7days. Anti-rabbit IgG IP was used as a negative control. (**C**) Anti-Runx2 IP in SIRT6-Tg VSMCs after treatment with Pi (3.0 mM) for 7days. Western blot was carried out with anti-SIRT6 or anti-Runx2 antibody. Anti-mouse IgG IP was used as a negative control. (**D**) The anti-HA IP and anti- flag IP followed by Western blot with anti-HA or anti-flag antibody in HEK-293T cells infected with HA-Runx2 plasmid, flag-SIRT6 plasmid, or both. Anti-rabbit IgG IP was used as a negative control. (**E**) WT and SIRT6-Tg VSMC lysates were immunoprecipitated with anti-Runx2 antibody and immunoblotted with anti-acetylated lysine antibody. (**F**) HEK-293T cells were infected with HA-Runx2 plasmid, flag-SIRT6 plasmid, or both. The anti-HA IP followed by Western blot with anti-acetylated lysine antibody and anti-HA antibody. (**G**) SIRT6-Tg VSMCs were pretransfected with siSIRT6 or siNC together with Pi (3.0 mM) for 7 days and OSS-128167 or DMSO were incubated with Pi (3.0 mM) for 7 days. The cell lysates were immunoprecipitated with anti-Runx2 antibody and immunoblotted with anti-acetylated lysine antibody and anti-Runx2 antibody. All the above experimental processing were duplicated 3 times.

We then sought to investigate the regulatory role of SIRT6 for Runx2. Quantitative PCR (qPCR) showed that Runx2 mRNA expression was not significantly changed between SIRT6-Tg and WT groups (Supplemental Figure 9D), which implied that SIRT6 had little impact on Runx2 transcription. Since SIRT6 is a NAD^+^-dependent deacetylase, we hypothesized that SIRT6 regulated Runx2 through influencing its acetylation status. As shown in IF staining assays, SIRT6 and Runx2 were colocalized in the nucleus of SIRT6-Tg VSMCs under Pi treatment (Figure 4A). We confirmed that SIRT6 physically interacted with Runx2 in co-IP assays (Figure 4, B and C) and this finding was further verified in human embryonic kidney (HEK) 293T cells transfected with HA-tagged Runx2 and Flag-tagged SIRT6 (Figure 4D).

We then assessed the acetylation level of Runx2. We found that Runx2 acetylation level decreased in SIRT6-Tg VSMCs compared with WT VSMCs under Pi treatment (Figure 4E). Similarly, the acetylation level of Runx2 was decreased in HEK-293T cells transfected with both Flag-SIRT6 and HA-Runx2 compared with cells transfected with HA-Runx2 alone (Figure 4F). Conversely, the Runx2 acetylation level was increased when silencing SIRT6 (Figure 4G). Taken together, these results suggested that SIRT6 deacetylated Runx2 in osteogenic transdifferentiation VSMCs.

### SIRT6 promotes Runx2 degradation via ubiquitin-proteasome system.

**Figure 5:**
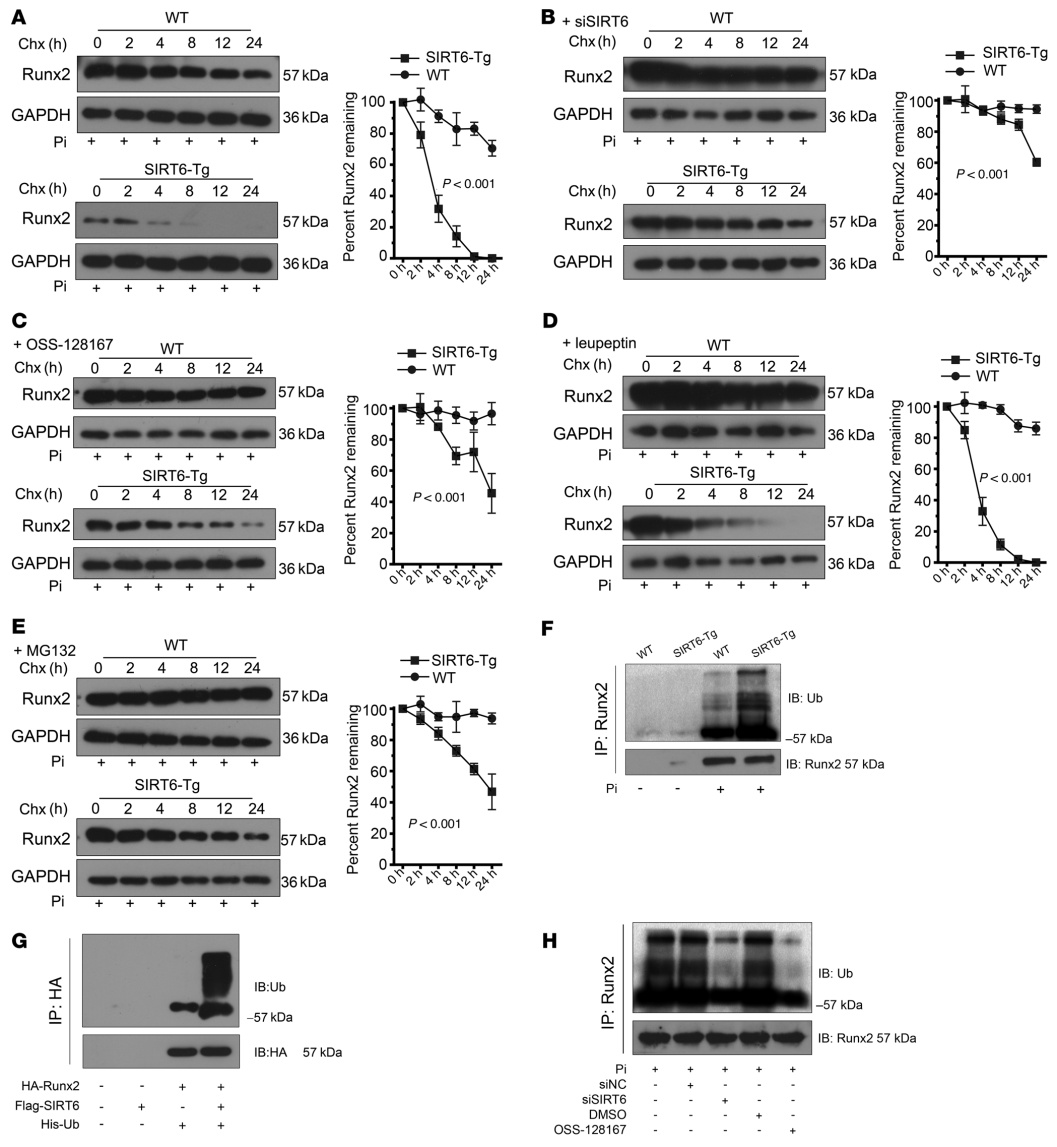
SIRT6 promotes Runx2 degradation via the ubiquitin-proteasome system. (**A**) WT and SIRT6-Tg VSMCs were treated with Pi (3.0 mM) for 7 days and incubated with the protein translation inhibitor CHX (0.2 mM) for the indicated times before harvest, followed by immunoblotting with the anti-Runx2 antibody and anti-GAPDH anti-body. The curve shows the stability of Runx2 protein. (**B** and **C**) SIRT6 was decreased in primary VSMCs via siRNA (**B**) or specific inhibitor (**C**) together with Pi (3.0 mM) incubation for 7 days. The protein translation inhibitor CHX (0.2 mM) was added for indicated times before harvest, followed by immunoblotting with the anti-Runx2 antibody and anti-GAPDH antibody. The curve shows the stability of Runx2 protein. (**D** and** E**) SIRT6-Tg VSMCs were incubated with Pi (3.0 mM) together with the leupeptin (1.5 μM) (**D**) or MG132 (10 μM) (**E**) for 7 days, and then the protein translation inhibitor CHX (0.2 mM) was added for the indicated times before harvest, followed by immunoblotting with the anti-Runx2 antibody and anti-GAPDH antibody. The curve shows the stability of Runx2 protein. (**F**) WT and SIRT6-Tg VSMC lysates were immunoprecipitated with anti-Runx2 antibody and immunoblotted with anti-ubiquitin (anti-Ub) antibody. (**G**) HEK-293T cells were transfected with His-Ub together with HA-Runx2 plasmid, flag-SIRT6 plasmid, or both. The anti-HA IP was followed by Western blot with anti-Ub antibody and anti-HA antibody. (**H**) SIRT6-Tg VSMCs were pretransfected with siSIRT6 or siNC together with Pi (3.0 mM) for 7 days, and OSS-128167 or DMSO were incubated with Pi (3.0 mM) for 7 days. The cell lysates were immunoprecipitated with anti-Runx2 antibody and immunoblotted with anti-Ub antibody and anti-Runx2 antibody. Statistical significance was assessed using 2-way ANOVA (**A**–**E**). All the above experimental processing was duplicated 3 times.

Since Runx2 acetylation was responsible for its stabilization (23, 24), we investigated if SIRT6 could influence Runx2 stabilization. The stability of Runx2 protein was reduced in SIRT6-Tg VSMCs after treatment with the protein synthesis inhibitor cycloheximide (CHX) (Figure 5A). Conversely, silencing SIRT6 prolonged the stability of Runx2 (Figure 5, B and C). In addition, SIRT6 protein stability didn’t show a significant change under Pi treatment (Supplemental Figure 10). To explore the manner of Runx2 degradation, the proteasome inhibitor MG132 and the lysosomal proteases inhibitor leupeptin were applied. As shown, leupeptin had no impact on Runx2 protein stability, but MG132 dramatically enhanced the protein stability of Runx2 in SIRT6-Tg VSMCs (Figure 5, D and E). These data indicated that SIRT6-induced Runx2 reduction was mediated by the proteasome but not the lysosome. Proteasome protein degradation often correlates with the specificity of target protein ubiquitin, and protein acetylation and ubiquitination are involved in the regulation of various cellular functions (25, 26). Therefore, we investigated the ubiquitination levels of Runx2 in SIRT6-Tg and WT VSMCs under Pi treatment. The ubiquitination level of Runx2 was upregulated in SIRT6-Tg VSMCs (Figure 5F). Similar results were observed in HEK-293T cells transfected with HA-Runx2 alone or together with Flag-SIRT6 (Figure 5G). In contrast, silencing SIRT6 resulted in a decrease of Runx2 ubiquitination in VSMCs (Figure 5H). Moreover, we further explored smad ubiquitin regulatory factor 1 (Smurf1) expression and its interaction with Runx2, since Smurf1 is a E3 ubiquitin ligase reported on degradation of Runx2. The results showed that there was less of a difference in Smurf1 expression between Pi-treated WT and SIRT6-Tg VSMCs. Interestingly, the interaction between Smurf1 and Runx2 was weaker in WT VSMCs than in SIRT6-Tg VSMCs (Supplemental Figure 11). These results further demonstrated that SIRT6 mediated the ubiquitination of Runx2 in VSMCs. Taken together, these data indicated that SIRT6 promoted Runx2 ubiquitination and subsequent proteasome-dependent degradation via Runx2 deacetylation.

### SIRT6 promotes Runx2 degradation through XPO1-dependent nuclear export.

**Figure 6:**
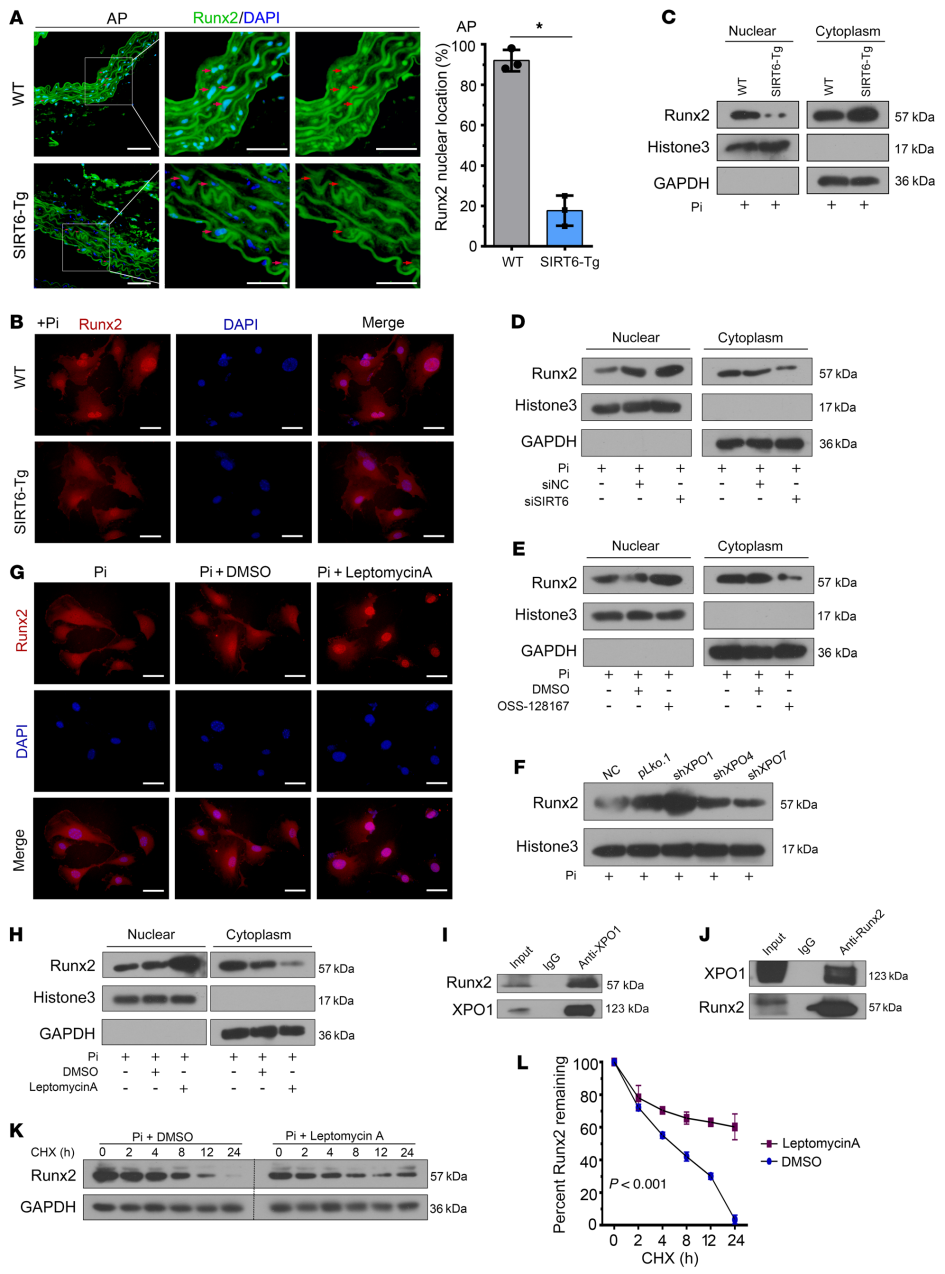
SIRT6 mediates Runx2 nuclear export depending on XPO1. (**A**) Runx2 IF staining was performed in abdominal arteries. Scale bar: 50 μm. Statistical significance was assessed using 2-tailed *t* tests, *P < 0.05. (**B**) VSMCs were incubated with Pi for 7 days. IF staining was performed for Runx2. Scale bars: 50 μm. (**C**) VSMCs were incubated with Pi for 7 days. Cells were harvested and immunoblotted for the indicated proteins. (**D**) SIRT6-Tg VSMCs were incubated with Pi for 7 days after posttransfection of siSIRT6. Cells were harvested and immunoblotted for the indicated proteins. (**E**) SIRT6-Tg VSMCs were incubated with Pi together with nicotinamide for 7 days. Cells were harvested and immunoblotted for the indicated proteins. (**F**) SIRT6-Tg VSMCs were transfected with shRNA targeting XPO1, XPO4, XPO7, or their vector control, and then incubated with Pi for 7 days after transfection. Nuclear extracts were immunoblotted for Runx2. (**G** and** H**) SIRT6-Tg VSMCs were incubated with Pi together with Leptomycin A (0.5 nM) for 7 days. Cells were harvested and immunoblotted for the indicated proteins (**G**). IF staining was performed for Runx2. Scale bars: 50 μm (**H**). (**I**) Anti-XPO1 IP followed by Western blot with anti-Runx2 or anti-XPO1 antibody in SIRT6-Tg VSMCs after treatment with Pi for 7 days. Anti-rabbit IgG IP was used as negative control. (**J**) Anti-Runx2 IP in SIRT6-Tg VSMCs after treatment with Pi for 7 days. Western blot was carried out with anti-XPO1 or anti-Runx2 antibody. Anti-mouse IgG IP was used as negative control. (**K**) SIRT6-Tg VSMCs were incubated with Pi together with Leptomycin A for 7 days, and then CHX (0.2 mM) was added for the indicated times before harvest, followed by immunoblotting for the indicated proteins. (**L**) Curve shows the stability of Runx2 and was assessed using 2-way ANOVA. Pi treatment is 3.0 mM. All the above experimental processing was duplicated 3 times.

A high Runx2 expression level was observed in calcified aorta from WT mice, while a low level was detected in SIRT6-Tg mice (Figure 6A). Interestingly, the nuclear accumulation of Runx2 was more abundant in WT VSMCs than in SIRT6-Tg VSMCs (Figure 6A). We explored whether the subcellular localization of Runx2 was related to SIRT6-mediated degradation. IF staining showed that nuclear accumulation of Runx2 was less predominant in SIRT6-Tg VSMCs under Pi treatment (Figure 6B). Similar results were found in immunoblotting analysis (Figure 6C). Conversely, nuclear accumulation of Runx2 was increased when silencing SIRT6 (Figure 6, D and E). These results demonstrated that SIRT6 modulated Runx2 subcellular localization in Pi-treated VSMCs.

It has been reported that importin β superfamily members exportin-1 (XPO1), exportin-4 (XPO4), and exportin-7 (XPO7) are related to protein nuclear export (27). Therefore, we knocked down these genes (Supplemental Figure 9B) to investigate their potential regulation of this process. Silencing XPO1, but not the other 2 members, abrogated the SIRT6-induced redistribution of Runx2 (Figure 6, F–H). Furthermore, we examined Runx2–XPO1 interaction by IP and found that Runx2 directly binds to XPO1 (Figure 6, I and J). Inhibiting XPO1 by leptomycin A treatment can prolong the stability of Runx2 in SIRT6-Tg VSMCs (Figure 6, K and L). Taken together, our data suggested that SIRT6-mediated Runx2 deacetylation resulted in redistribution of Runx2 through XPO1.

### SIRT6 impedes vascular calcification depending on nuclear export of Runx2.

**Figure 7:**
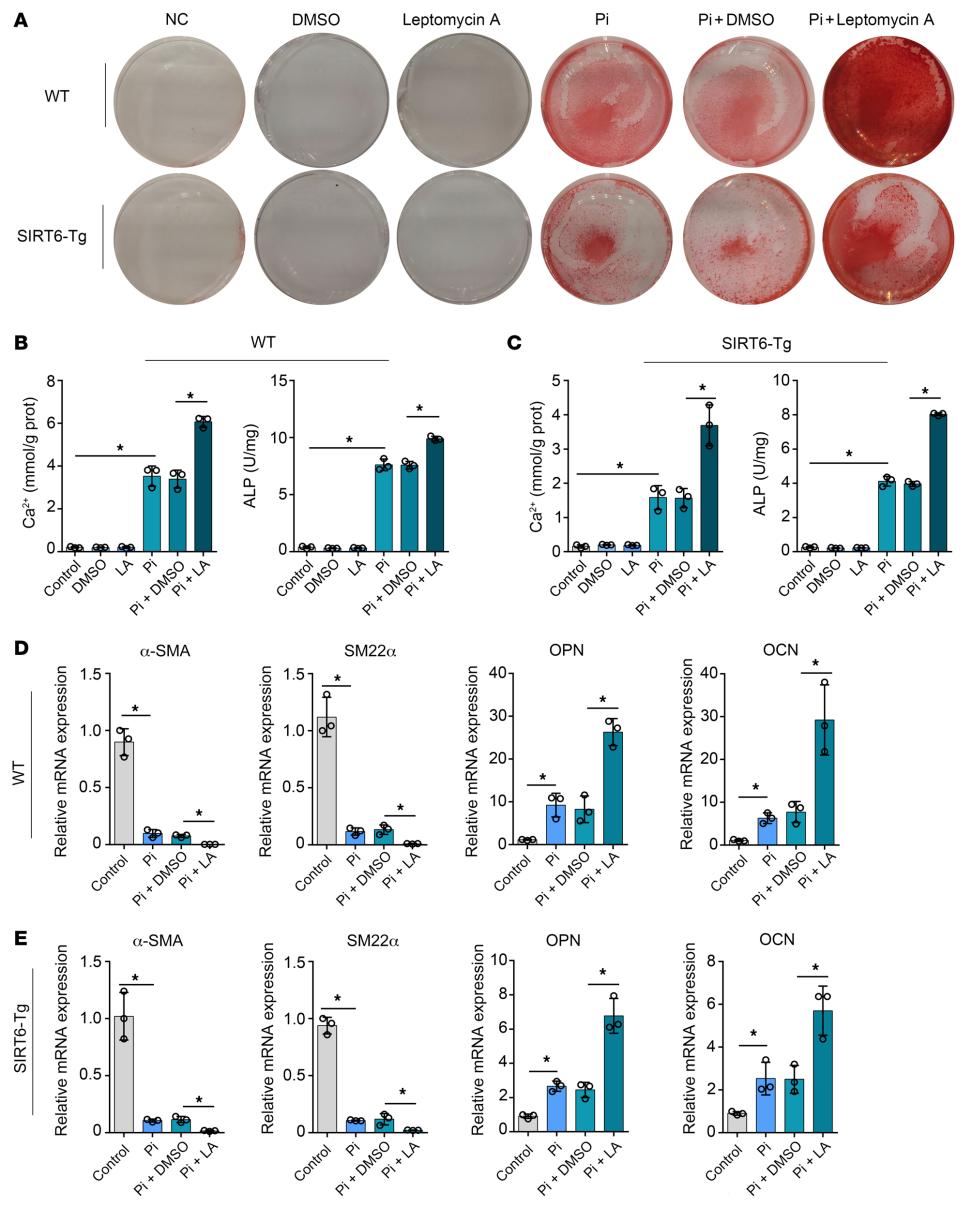
Nuclear export of Runx2 is a key component of SIRT6 vascular calcification suppressor function. (**A**–**C**) WT and SIRT6-Tg VSMCs were incubated with Pi (3.0 mM) together with Leptomycin A for 7 days. VSMCs were stained for mineralization by Alizarin red S (**A**), and calcium content (**B**) and ALP (**C**) were quantified (*n =* 3 per group). (**D** and** E**) The osteogenic markers (OPN, OCN) and the contractile property markers (α-SMA, SM22α) were analyzed by qPCR for the WT (**D**) and SIRT6-Tg VSMCs (**E**) mouse being incubated with Pi (3.0mM) together with Leptomycin A for 7 days (*n =* 3 per group). Statistical significance was assessed using 1-way ANOVA followed by Dunnett’s test (**B**–**E**). *P < 0.05. All values are mean ± SD.

We performed additional experiments to confirm the nuclear export role of XPO1 in VC attenuation mediated by SIRT6. As expected, XPO1 inhibitor treatment significantly increased calcium deposition in both SIRT6-Tg and WT VSMCs (Figure 7, A–C). Similarly, Leptomycin A inhibition of XPO1 reversed the suppressive role of SIRT6 in osteogenic transdifferentiation of VSMCs (Figure 7, D and E). Based on these findings, we concluded that XPO1 played a critical role in SIRT6-mediated VC attenuation.

## Discussion

In this study, we elucidated a novel SIRT6/Runx2 pathway in vascular calcification. For the first time, we found that SIRT6 suppressed VSMC osteoblastic transdifferentiation and attenuated VC both in vivo and in vitro. Mechanistically, SIRT6 deacetylated Runx2 and promoted its ubiquitination and subsequent degradation through the ubiquitin-proteasome system.

There are 7 sirtuins (SIRT1-7) in mammals and each family member has a different function and subcellular localization. The common molecular targets suggest that sirtuins might act synergistically. Here, using VSMC calcification in vitro, we showed that all members of the sirtuin family except SIRT4 are expressed in VSMCs. It’s known that SIRT1 is implicated in the transcriptional and epigenetic modifications of cellular and systemic processes. SIRT1 has proved to act in a protective role against VC (28, 29). However, SIRT1 modulators have not seen marked results in clinical studies (13). In this study, we found that only SIRT6 not SIRT1 was significantly downregulated at different calcification levels. The result indicated that SIRT6 played a critical role in VC.

SIRT6 is mainly located in the nucleus, and it is a class IV sirtuin that exhibits deacetylase and ADP-ribosyltransferase activity. SIRT6 is known to exert a protective role in atherogenesis and ischemic stroke, and act against VSMC differentiation in response to the cyclic strain (30–32). SIRT6 plays a role in a variety of biological processes, and it is responsible for a set of age-related disorders (33). CKD is one of the most typical age-related metabolic diseases. However, the association between SIRT6 and VC in CKD remains unknown. Using 2 canonical CKD models (adenine-induced and 5/6 nephrectomy-induced CKD mice), we reported that VC was less prominent in SIRT6-Tg mice than the WT controls. And SIRT6 prevented VC of VSMCs induced by Pi in vitro. VSMC-specific, SIRT6 knock down of aorta by AAV2 caused severe VC in the WT mouse model. In our clinical study, a lower expression level of SIRT6 was observed in calcified radial arteries and PBMCs of patients with CKD with VC. No significant differences were observed in kidney function or traditional risk factors between those with or without VC. Thus, these findings indicated that SIRT6 may act as a protective regulator in vascular calcification and its protective effect was independent of renal function changes.

Previous studies have demonstrated that the phenotypic transdifferentiation of VSMCs, from contractile to osteochondrogenic, is a pro-calcifying process and appears to initiate before mineral deposition (9, 10, 34). During this process, the osteoblastic features of VSMCs predominate, with decreased expression of contractile proteins (α-SMA and SM22α) and increased levels of the synthetic proteins (OPN and OCN). We investigated the effect of SIRT6 on phenotypic transdifferentiation of VSMCs. SIRT6 can reverse protein expression and mRNA level of α-SMA and SM22α and reduce protein expression and mRNA transcription of synthetic proteins such as OPN and OCN during the process of VC. Thus, the protective role of SIRT6 in VC attenuation was potentially mediated by inhibiting the phenotypic transdifferentiation of VSMCs. Upregulation of Runx2 expression has been observed in vascular calcification and its core role in VSMCs osteochondrogenic differentiation has been well documented (35–38). Posttranslational modifications of Runx2 can influence its stability and transcriptional activity. Runx2 can be phosphorylated by Erk/MAPK (24) and Akt (39). In atherosclerotic calcification, AMPKα1 promotes Runx2 SUMOylation, decreasing its stability (40). PTEN/AKT also modulated Runx2 ubiquitination via phosphorylating FOXO1/3 in VSMC calcification (41). In addition, enhancing acetylation of Runx2 promotes its stability and transcriptional activity (42–44).

We found that protein expression of Runx2 was significantly decreased in a SIRT6 overexpression VC model in vivo and in vitro. Enhancing Runx2 expression via plasmid reversed the protective effect of SIRT6 in vitro. This indicated Runx2 was regulated by SIRT6. The transcription level of Runx2 was not significantly affected by SIRT6, so we hypothesized that posttranslational regulation of Runx2 may be involved. Emerging evidence has shown that among the posttranslational modifications of Runx2 (24, 26, 45–47), SIRT6 is a deacetylase that could deacetylate the lysine residues of histone and nonhistone substrates, which is closely related to protein degradation via ubiquitination (48, 49). It has been reported that acetylation of Runx2 plays an important role in osteogenesis (50). Here, we found that Runx2 acetylation was reduced in VSMCs with SIRT6 overexpression, and identified physical interaction between Runx2 and SIRT6 proteins via co-IP assay. At the same time, reduction of Runx2 protein in the SIRT6 overexpression group was attributed to a shorter half-life. Normally, the acetylation of Runx2 could protect against the ubiquitin-proteasome degradation process (26). Inhibition of the proteasome via MG132 prevented SIRT6-mediated downregulation of the Runx2 protein. As expected, the ubiquitinated Runx2 was increased in SIRT6-Tg VSMCs, and the ubiquitinated Runx2 was almost abrogated in SIRT6-deficient WT VSMCs. These data indicated that SIRT6 was vital for ubiquitin-dependent proteolysis of Runx2. As reported in previous studies, Smurf1-mediated degradation of Runx2, and Runx2 acetylation, inhibited this interaction. We also found that the combination/interaction of Smurf1 and Runx2 was weaker in WT VSMCs than in SIRT6-Tg. Collectively, these data suggested that SIRT6 deacetylates Runx2, which was subsequently ubiquitinated, and degraded through the proteasome.

Runx2 undergoes diverse posttranslational modifications, some of which may regulate its subcellular distribution, and nuclear-cytoplasmic shuttling of Runx2 may regulate cell fate (51). However, the subcellular distribution of Runx2 has not been explored in VC. Our data suggested that nuclear levels of Runx2 were higher in WT than in SIRT6-Tg VSMCs. There was an increase in the Runx2 nuclear fraction under SIRT6 deficiency. We explored the Runx2 nuclear export mechanism and identified XPO1 as the specific transporter, in accordance with studies that reported that XPO1 regulated Runx2 nuclear-cytoplasmic shuttling (51, 52). Our in vitro VC models revealed that the XPO1 was vital for SIRT6-mediated attenuation of VC and we also observed that reduced nuclear export of Runx2 can prolong its half-life. These findings demonstrated a unique mechanism of Runx2 degradation, which was mediated through deacetylation-dependent Runx2 nuclear export.

Our previous study demonstrated alkB homolog 1 (Alkbh1) upregulation on the progression of VC via activation of the osteogenic protein, bone morphogenetic protein 2 (BMP2; ref. 53).Runx2 was a major target of BMP2 pathway and BMP2 was proved to regulate the acetylation and ubiquitination level of Runx2 (26, 43). Thus, BMP2 and Runx2 cooperatively interact to induce VC. These indicate that SIRT6 upregulation may play an important role against the BMP2 pathway in VC. In addition, further studies are required to demonstrate the exact regulatory effect of Alkbh1/BMP2 pathway on SIRT6 expression in VC. SIRT6 is known for improving longevity, modulating genome stability and telomere integrity, and reducing oxidative stress and inflammation (14, 33, 54). It has also been reported that Runx2 negatively regulates SIRT6 expression at both the transcriptional and posttranslational levels in breast cancer (55). In our results, we found that Runx2 did not play a role in regulation of SIRT6 expression in VSMCs (neither in transcription nor posttranslation). SIRT6 expression was significantly associated with disease status of blood vessels, and SIRT6 expression data from PBMCs can be used as a disease marker for predicting calcification in patients with CKD. Further studies are needed to demonstrate the relationship between PBMCs and VSMC calcification.

Collectively, our studies demonstrate for the first time that SIRT6 prevents VC through posttranslational regulation of Runx2 activity and stability. These findings suggest that SIRT6 may be an innovative therapeutic strategy for VC.

## Methods

### CKD patient samples.

Peripheral blood samples from patients with CKD and healthy people were collected from Donghua Hospital of Sun Yat-sen University from November 2019 to January 2020. Thirty-nine patients with CKD and 20 healthy people were recruited to this study. PBMCs from peripheral blood were extracted using Histopaque-1077 (Sigma) gradient. The extract mixture was centrifuged at 400*g* for 20 minutes and the interface was collected as PBMCs. Clinical and biochemical parameters were collected from the patient electronic medical records in the hospital. The radial arteries from patients with CKD undergoing hemodialysis were collected from The Eighth Affiliated Hospital of Sun Yat-sen University from November 2019 to January 2020.

### Assessment of thoracic aorta calcification score.

Patients underwent a chest multi-detector computed tomography (MDCT) scan with standard electrocardiographically (ECG) gated protocol to evaluate thoracic aorta calcification. Agatston scores of images were blind-quantified by 3 independent investigators with Siemens Syngo CT Workplace software according to standard criteria (56). The thoracic aorta refers to the section between the ascending and descending aorta. To measure calcification scores, the CT images were reconstructed with 1 mm–thick slices. The presence of calcification was defined as Agatston score in the present study.

### Induction of VC in mice.

Male mice were used in this study to avoid the potential interference of changing levels of hormones on VC. WT C57BL/6J mice at 8 weeks and weighing 25 to 30 grams were purchased from the Laboratory Animal Center of Sun Yat-sen University. Cloned mSirt6 cDNA with CAG promoter was injected into fertilized eggs to constructed Sirt6-transgenic mice (SIRT6-Tg) of C57BL/6J background as was previously reported (57). The phenotype of SIRT6-Tg mice and genotyping identification procedure were identified by One Step Mouse Genotyping Kit (Vazyme) according to the manufacturer’s instructions. Tail DNA was used to confirm mice positive for the transgene at 2 to 3 weeks of age. The following primers were used for genotyping: forward, 5′-GCCGTCTGGTCATTGTCAACCTG-3′; reverse, 5′-AAAGACCCCTAGGAATGCTCGTCAA-3′. Eight-week-old SIRT6-Tg mice weighing 25 to 30 grams were used for these experiments. All mice were raised in the Laboratory Animal Center of Sun Yat-sen University and were maintained in a temperature-controlled room on a 12-hour light/dark cycle with available access to food and water. WT and SIRT6-Tg mice were randomly assigned to experimental groups with at least 12 animals in each group: the control group was fed with standard pellet chow diet (normal diet, ND) and the CKD model group was fed with special chow containing 0.75% adenine and high (1.5%) levels of phosphorus (AP diet) or performed a 5/6 nephrectomy model. After 12 weeks of AP diet or 8 weeks of high phosphorus diet after 5/6 nephrectomy, the animals were analyzed to confirm the vascular calcification of aorta, and then sacrificed. The aorta was harvested from each animal and was kept at –80°C for further use. The VC Agatston scores of aortas were analyzed by 3 independent investigators and the score was normalized to the lowest score (not zero) in SIRT6-Tg group. For VSMC-specific SIRT6 knockdown, the WT mice were injected in the lateral tail vein with recombinant AAV serotype 2 gene transfer vectors bearing a VSMC-specific promoter combination (SM22α promoter) with mouse sh-SIRT6 sequence. After 4 weeks, some of the mice were sacrificed and aortas and kidneys were collected. Western blot was used to confirm the efficiency of AAV-sh-SIRT6 in aortas and kidneys. The remaining mice were treated with AP diet for 12 weeks, or a 5/6 nephrectomy was performed and then mice were fed with high phosphorus diet for another 8 weeks. Then the mice were sacrificed and aortas were collected. The detailed protocols were shown in our previous study (53). The AAV2 was generated by Hanbio.

### Cell culture.

Primary HAoSMCs were purchased from ATCC and cultured in DMEM containing 10% FBS supplemented with 100 U/mL penicillin, 100 μg/mL streptomycin.

Mice VSMCs were isolated from 6-week-old SIRT6-Tg mice and WT C57BL/6J control mice. Briefly, the adventitia and endothelium were removed from the thoracic aortic arteries and the remaining tissue was cut into approximately 1 mm^2^ sections. Aorta segments were placed in cell culture dishes with DMEM containing 10% FBS, 100 U/mL penicillin, and 100 μg/mL streptomycin in a 37°C incubator with 5% CO_2_ for 5 to 7 days. The VSMCs migrated from the explants, and cells between passages 5 and 8 were used in experiments.

### VSMCs calcification induction.

To induce calcification, VSMCs at 80% confluence were incubated in DMEM containing 10% FBS, 100 U/mL penicillin, and 100 μg/mL streptomycin, with the addition of 3.0 mmol/L sodium phosphate (Pi) (Sigma) and cultured at 37°C in an incubator containing 5% CO_2_ for 7 days. The medium and Pi were refreshed every 2 days. The control VSMCs were treated with DMEM containing 10% FBS, 100 U/mL penicillin, and 100 μg/mL streptomycin, but without Pi, and the medium was also refreshed every 2 days.

### von Kossa assay.

To examine aorta calcification, slides were dehydrated and rinsed rapidly in double distilled water. The vascular tissue sections were then incubated with 5% silver nitrate solution and exposed to ultraviolet light for 1 hour until color development was complete. Next, the slides were incubated with 5% sodium thiosulfate and washed with double distilled water. The slides were photographed by microscopy (Nikon). Calcified nodules were stained brown to black.

### Alizarin red staining.

At collection time points, medium was removed and cultured VSMCs were washed with 4°C PBS 3 times (3 minutes each wash), and then cell layers were fixed in 4% paraformaldehyde in PBS for 20 minutes. Next, the paraformaldehyde was removed and the cells were washed in distilled water 3 times (2 minutes each wash). The cells were then exposed to Alizarin red staining solution (pH 4.2, 1%) for 30 minutes at room temperature, then washed again with distilled water. Positively stained VSMCs presented a reddish color to indicate the calcification.

### Calcium and ALP quantification.

Aortic tissues without adventitia were incubated with 0.6 mol/L HCl overnight at 37°C. The supernatant of these tissues was then collected. The cultured VSMCs were washed softly with PBS for 3 times (2 minutes each wash) and incubated with 0.6 mol/L HCl overnight at 4°C. The supernatant was collected. Calcium content was determined by using a commercial kit (Biosino Bio-Technology and Science) according to the manufacturer’s instructions. VSMCs or aortic tissues were equilibrated with 1% Triton X-100 in 0.9% saline on ice and the supernatant was collected for ALP quantification assay after centrifugation in a microfuge at 8000*g* for 5 minutes. ALP activity was analyzed using a commercial assay kit (Biosino Bio-Technology and Science). Results are shown normalized to total protein levels.

### Quantitative real-time PCR.

Total RNA was extracted from aortic tissue and VSMCs by using Trizol Reagent (Takara) according to the manufacturer’s instructions. For mRNA quantification, a PrimeScriptRT Reagent Kit (Takara) was used for RNA reverse transcription into cDNA. Real-time PCR was performed with SYBR Green (Takara) and data were collected and analyzed using a LightCycler 96 real-time system (Roche Diagnostics). Relative quantification was calculated according to the 2^ΔΔCt^ method, with GAPDH level as a reference. The primer sequences are listed in Supplemental Table 1.

### Transfection and transduction of VSMCs and HEK-293T cells.

For siRNA and shRNA transfection, VSMCs were plated at 5 × 10^5^ cells in 6-well plates. At 50% confluence, cells transfected with specific siRNA at a final concentration of 10 nmol/L with Lipofectamine 3000 (Invitrogen) according to the manufacturer’s instructions. After 6 hours of transfection with opti-MEM, the DMEM containing 10% FBS was replaced. The full-length of the target gene cDNA was amplified from a mouse cDNA library using standard PCR techniques and inserted into pcDNA3.1. For plasmid transfection, cultured VSMCs or HEK-293T cells were transfected with specific plasmids by Lipofectamine 3000 regent according to the manufacturer’s instructions. The relative siRNA and shRNA are listed in Supplemental Table 2.

### Immunofluorescence staining and immunohistochemistry.

The VSMCs were first washed with 1× PBS 3 times, and then fixed with 4% paraformaldehyde solution for 20 minutes. Next, the paraformaldehyde was removed and cells were washed in PBS 3 times. Cells were permeabilized using 0.1% Triton-X. After another 3 PBS washes, cells were incubated with 5% BSA for 30 minutes. Following this, the primary antibody for rabbit anti-SIRT6 (Abcam) or mouse anti-Runx2 (Abcam) was incubated overnight at 4°C. FITC-labeled (Sigma) or Alexa Fluor 647–labeled secondary antibodies (Abcam) were incubated for 1 hour at room temperature. DAPI (Solarbio) for staining nuclei was incubated for 5 minutes at room temperature and then cells were washed in PBS 3 times. Imaging was performed using Olympus IX73fluorescence microscope (Olympus). The antibody details can be found in Supplemental Table 3.

Radial arteries from hemodialysis patients and mice aortic tissues were formalin-fixed and further embedded with paraffin. For immunostaining, tissue sections were deparaffinized in xylene and rehydrated through a graded alcohol series to distilled water. Antigen retrieval was performed by microwave irradiation in ethylene diamine tetraacetic acid (EDTA). Then tissue sections were incubated with 5% normal goat serum in PBS/0.1% Triton X-100 for 1 hour at room temperature to reduce nonspecific background staining. Sections were then incubated overnight at 4°C with primary antibody for rabbit anti–α-SMA (Abclone), rabbit anti-OPN (Proteintech) or rabbit anti-Runx2 (CST). For IF, binding of primary antibodies was visualized using goat anti-rabbit FITC-labeled antibody incubated for 1 hour at room temperature. Nuclei were counterstained with DAPI. Prolong Gold antifade reagent was used to decrease fluorescence quenching of the slides. For IHC, expression of SIRT6 in WT and SIRT6-Tg mouse was stained with SIRT6 antibody by universal SP kit (ZSGB-BIO) according to the manufacturer’s instructions. The images were collected with an Olympus IX73 fluorescence microscope. The primary antibodies are listed in Supplemental Table 3.

### Nuclear/cytoplasmic extraction.

At collection time points, culture medium was removed and then VSMCs were washed with 1× PBS for 3 times. The nuclear and cytoplasmic protein lysate extraction of VSMCs was performed using the Nuclear Protein Extraction Kit (Solarbio) according to the manufacturer’s recommendations.

### Immunoprecipitation and Western blot analysis.

Harvested VSMCs and HEK-293T cells were lysed with lysis buffer (Beyotime) together with protease and phosphatase inhibitors on ice for 15 minutes. The lysate was then sonicated on ice at 10% power for 2 minutes. After centrifugation at 12,000*g* for 20 minutes at 4°C, the supernatant was precleared by incubation with protein A+G magnetic beads (Millipore) and IgG (CST) for 1 hour at 4°C. The samples were then place in a magnetic separator for 1 minute. The supernatant was incubated with indicated antibody overnight at 4°C on a rotating platform. Protein A+G magnetic beads were then added to the supernatants and incubated for 2 hours at room temperature. The immunocomplexes were washed 3 times with the lysis buffer, boiled at 95°C for 10 minutes with 2× SDS sample buffer, and analyzed by Western blot. For Western blot analysis, the cells lysates or tissue pieces were prepared by adding the lysis buffer on ice for 15 minutes, supplemented with protease and phosphatase inhibitors, scraping into a 1.5 mL tube, and centrifuging for 20 minutes at 12,000*g* at 4°C. The protein content was measured by enhanced BCA protein assay kit (Beyotime). The proteins were boiled in loading buffer (Beyotime) at 100°C for 10 minutes. Equal amounts of proteins were separated on SDS-polyacrylamide gels and transferred to PVDF membranes (Millipore). The membranes were incubated with the primary antibodies overnight at 4°C. The membranes were then incubated with secondary anti-rabbit (CWBIO) or anti-mouse (CWBIO) HRP-conjugated antibody (diluted 1:10,000) for 1 hour at room temperature. Antibody binding was detected with ECL detection reagent (Millipore). The relative quantification of immunoblots was analyzed by grayscale in ImageJ. The antibodies used in this study are listed in Supplemental Table 3.

### Statistics.

All data are mean ± SD. Statistical analyses were performed with the Graphpad Prism v6.00 for Windows (GraphPad Software Inc.). Student’s *t* test was used to compare 2 groups and 1-way ANOVA followed by Dunnett’s test was used for more than 2 groups. VC Agatston scores were nonnormalized parameters, and logarithmic transformation of VC Agatston scores was used in correlation analysis (Pearson Correlation Analysis). Statistical significance was accepted at *P* less than 0.05.

### Study approval.

All the related procedures for collection of the samples of patients with CKD and normal people were performed with the approval from the internal review and ethics board of Donghua Hospital of Sun Yat-sen University and The Eighth Affiliated Hospital of Sun Yat-sen University. All participants signed informed consent before entering this study. Experimental animal protocols were approved by the Institutional Animal Care and Use Committee of Sun Yat-sen University.

## Author contributions

HH conceived the project. WL, WF, ZL, and BL performed and analyzed in vivo experiments. WL, WF, and Y Zhou performed the in vitro experiments and analyzed the data. XS and MZ performed and analyzed the biochemical and biophysical experiments. DL and Y Zhu were responsible for human clinical and molecular genetic studies. WL, DL, and JC wrote the paper with input from all authors. The images were photographed by WL. The order of co–first authors was determined by their efforts and contributions to the manuscript.

## Supplementary Material

Supplemental data

## Figures and Tables

**Table 1:**
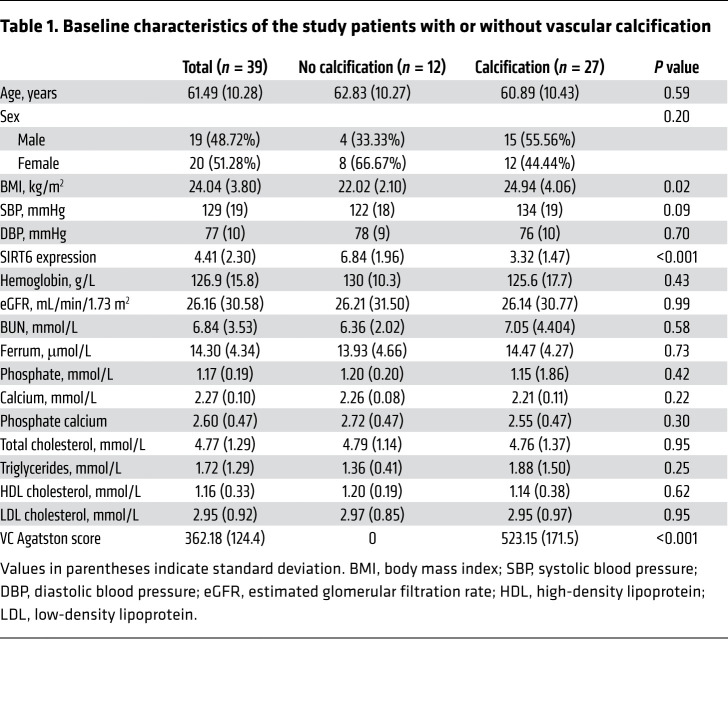
Baseline characteristics of the study patients with or without vascular calcification

## Version 1 11/18/2021

In-Press Preview

## Version 2 01/04/2022

Electronic publication
