# Supplementary material for: SIRT6 protects vascular smooth muscle cells from osteogenic transdifferentiation via Runx2 in chronic kidney disease
Source: J Clin Invest. 2022 Jan 4;132(1):e150051. doi: 10.1172/JCI150051 (PMC8718147; doi:10.1172/JCI150051)
Supplement: Supplemental data [file jci-132-150051-s083.pdf]

SIRT6 protects vascular smooth muscle cell from osteogenic transdifferentiation via Runx2 in chronic kidney disease

Wenxin Li[1]; Weijing Feng[2]; Xiaoyan Su[3]; Dongling Luo[1] ; Zhibing Li[4]; Yongqiao Zhou[4]; Yongjun Zhu[1]; Mengbi Zhang[3]; Jie Chen[5]; Baohua Liu[6]; Hui Huang[1]

1. Department of Cardiology, The Eighth Affiliated Hospital, Sun Yat-sen University, No.3025, Shennan Middle Road, Futian District Shenzhen 518000, China. 2. Department of Cardiology, State Key Laboratory of Organ Failure Research, Guangdong Provincial Key Lab of Shock and Microcirculation, Nanfang Hospital, Southern Medical University, Guangzhou 510280, China. 3. Nephropathy Department, Tungwah Hospital of Sun Yat-sen University Dongguan 523110, China. 4. Department of Cardiology, Sun Yat-sen Memorial Hospital, Sun Yat-sen University, Guangzhou 510120, China. 5. Department of Radiation Oncology, Sun Yat-sen Memorial Hospital, Sun Yat-sen University, Guangzhou 510120, China. 6. Shenzhen Key Laboratory for Systemic Aging and Intervention, National Engineering Research Center for Biotechnology (Shenzhen), Shenzhen University Health Science Center, Shenzhen, China

Address correspondence to:

Professor Hui Huang
Department of Cardiology
The Eighth Affiliated Hospital
Sun Yat-sen University
No.3025, Shennan Middle Road
Futian District Shenzhen 518000
China
huangh8@mail.sysu.edu.cn

Athorship note: WL, WF, XS and DL are co–first authors.

Conflicts of interest: The authors have declared that no conflict of interest exists.

Supplemental Materials

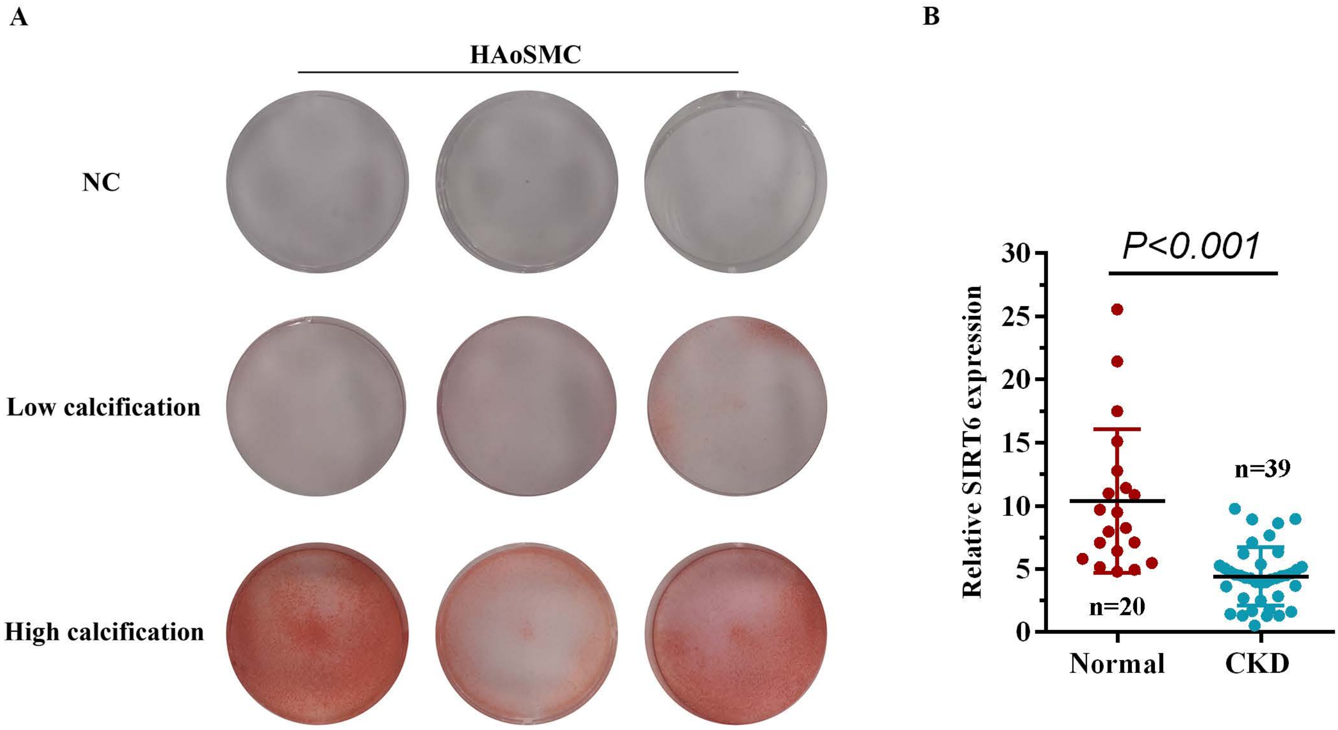

Supplemental Figure 1

A. The HAoSMCs was incubated without or with Pi (1.0 mM and 3.0 mM) to induce different calcified status. HAoSMCs exhibited low calcification in 1.0 mM Pi while high calcification in 3.0 mM Pi. **B**. SIRT6 mRNA levels in PBMCs from normal people with (n = 20) and CKD patients (n=37). Data is expressed as mean ± S.D., calculated using two-tailed t-test.

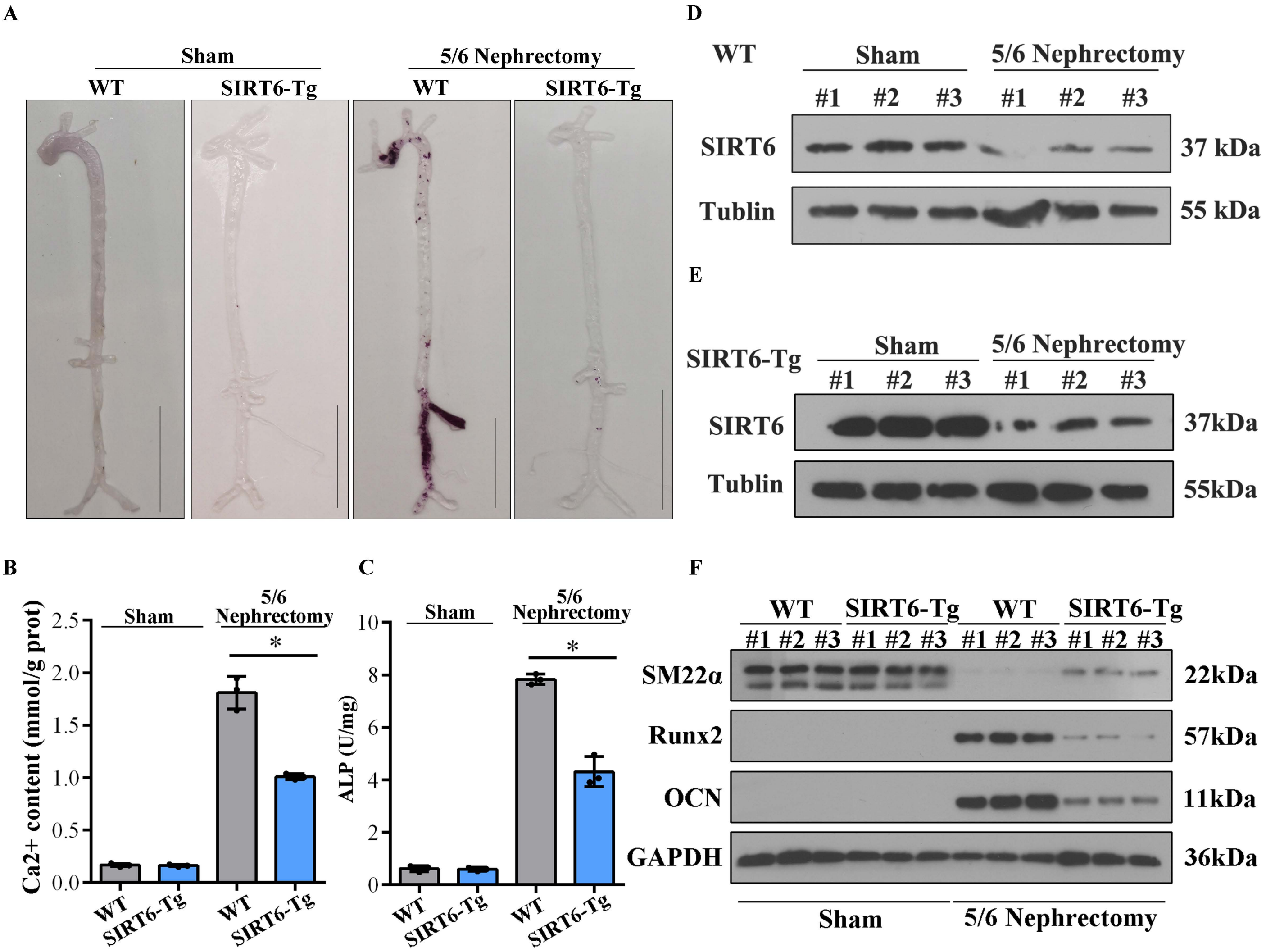

Supplemental Figure 2

A. Alizarin red S images showing calcification in aortas of 5/6 nephrectomy CKD model. The calcification of aorta were shown in deep purple. Scale bars, 10 mm. **B, C.** The SIRT6 expression of aortas between sham and 5/6 nephrectomy CKD mouse model in WT and SIRT6-Tg mice. **D, E.** Calcium content (**D**) and ALP (**E**) were quantified in aortas tissue between sham and 5/6 nephrectomy CKD mouse model in WT and SIRT6-Tg mice. **F.** Analysis of osteogenic and contractile property factor expression of aortas tissue between sham and 5/6 nephrectomy CKD mouse model in WT and SIRT6-Tg mice by Western blot. Data are expressed as mean ± SD., calculated using one-way ANOVA followed by Dunnett's test. $^*P < 0.05$.

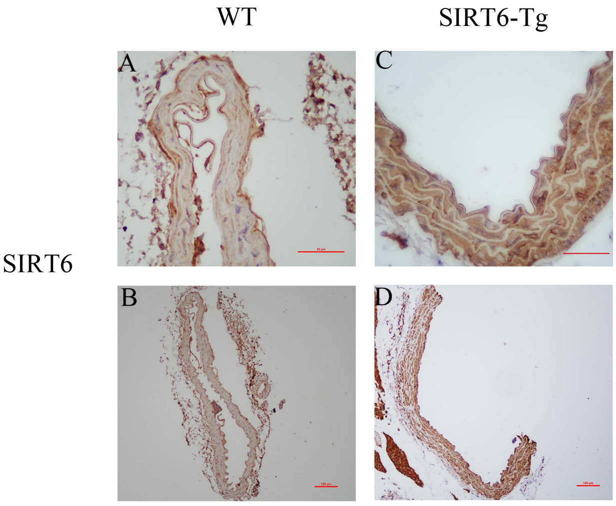

Supplemental Figure 3

SIRT6 expression in aorta of WT (A, B) and SIRT6-Tg mouse (C, D) detected by IHC. Scale bars: A, C =100µm； B, D = 50µm.

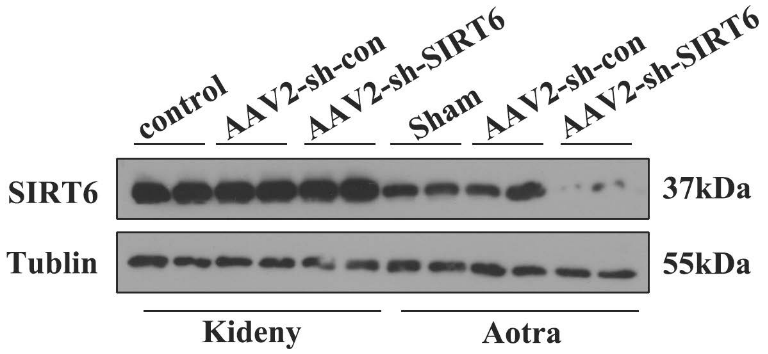

Supplemental Figure 4

WT mice were treated by AAV-sh-control and AAV-sh-SIRT6 for 4 weeks. Then the mice were sacrificed and collected aortas and kidneys. The SIRT6 expression among these groups were detected by Western blot to confirm the efficiency of AAV-sh-SIRT6 in aorta and kidney.

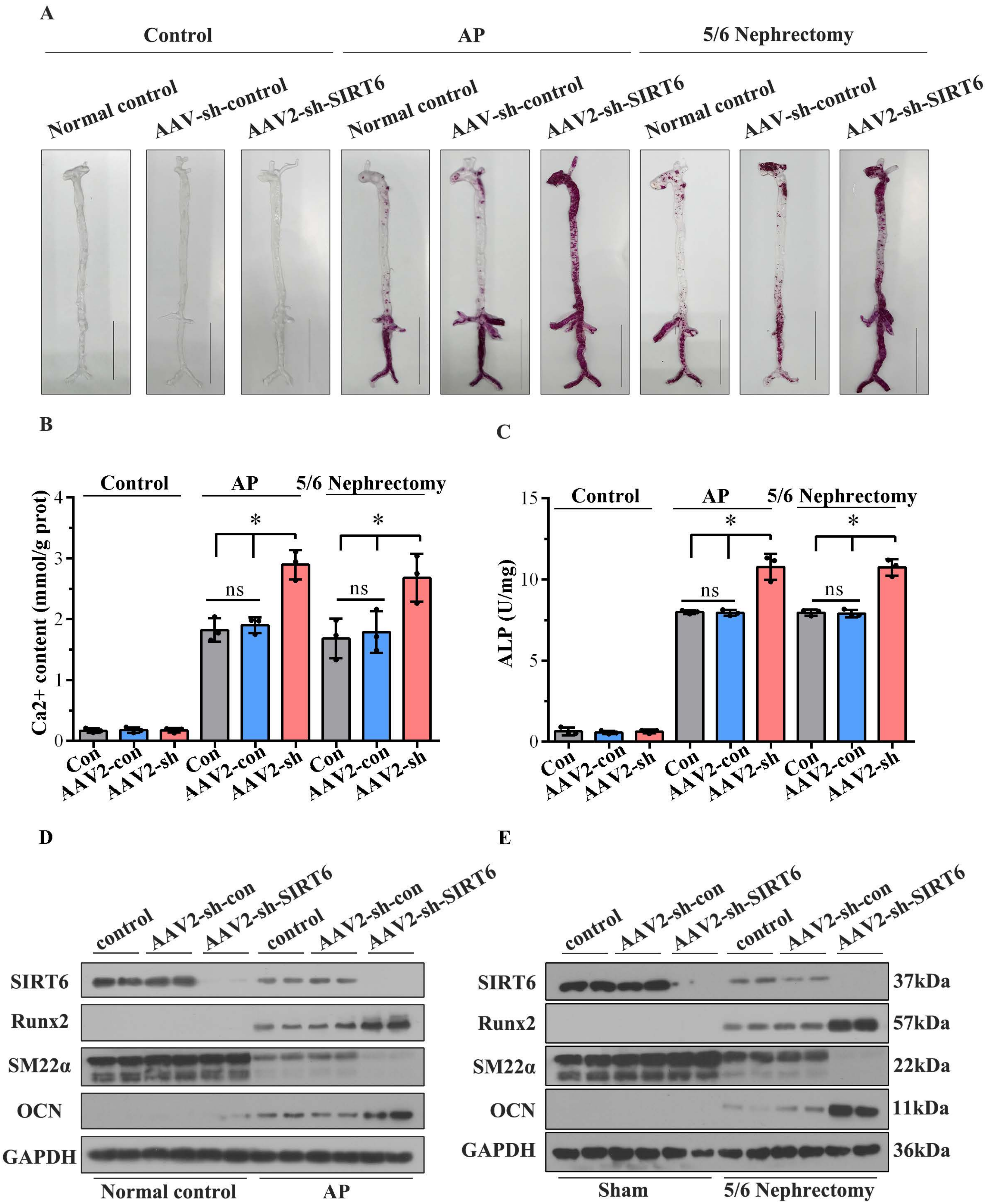
A
Control
AP
5/6 Nephrectomy
Normal control
AAV-sh-control
AAV2-sh-SIRT6
Normal control
AAV-sh-control
AAV2-sh-SIRT6
Normal control
AAV-sh-control
AAV2-sh-SIRT6
B
Control
AP
5/6 Nephrectomy
Ca2+ content (mmol/g prot)
ns
ns
*
*
Con
AAV2-con
AAV2-sh
C
ALP (U/mg)
D
control
AAV2-sh-con
AAV2-sh-SIRT6
SIRT6
Runx2
SM22α
OCN
GAPDH
Normal control
AP
E
37kDa
57kDa
22kDa
11kDa
36kDa
Sham
5/6 Nephrectomy

Supplemental Figure 5

A. WT mice were treated by AAV-sh-control and AAV-sh-SIRT6 for 4 weeks. Then these mice were treated with adenine and phosphorus diet (AP) for 12 weeks, or performed 5/6 nephrectomy and fed with high phosphorus for another 8 weeks. Then the mice were sacrificed and collected aortas. Alizarin red S images showing calcification in aortas among these groups. The calcified parts of aorta were shown in deep purple. Scale bars, 10 mm. **B, C.** Calcium content (**B**) and ALP (**C**) were quantified in aortas tissue among these groups. **D, E.** Analysis of osteogenic and contractile property factor expression of aortas tissue among these groups by Western blot. Data are expressed as mean ± SD., calculated using one-way ANOVA followed by Dunnett's test. *P < 0.05.

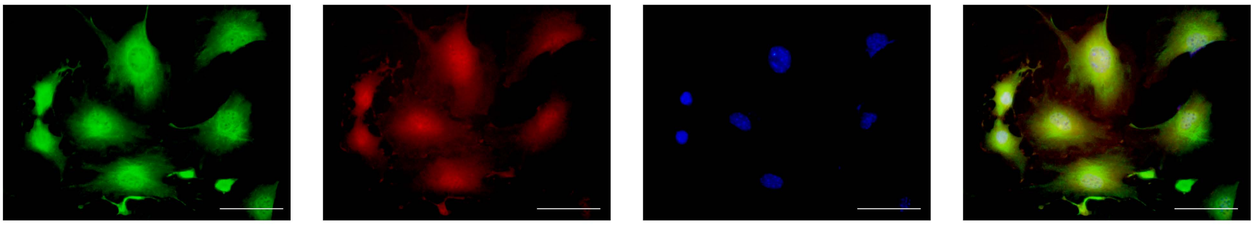

Supplemental Figure 6

The primary cells isolated from mouse aorta was identified by VSMCs marker (smooth muscle myosin heavy chain and SM-22α) in IF. Scale bar, 50μm

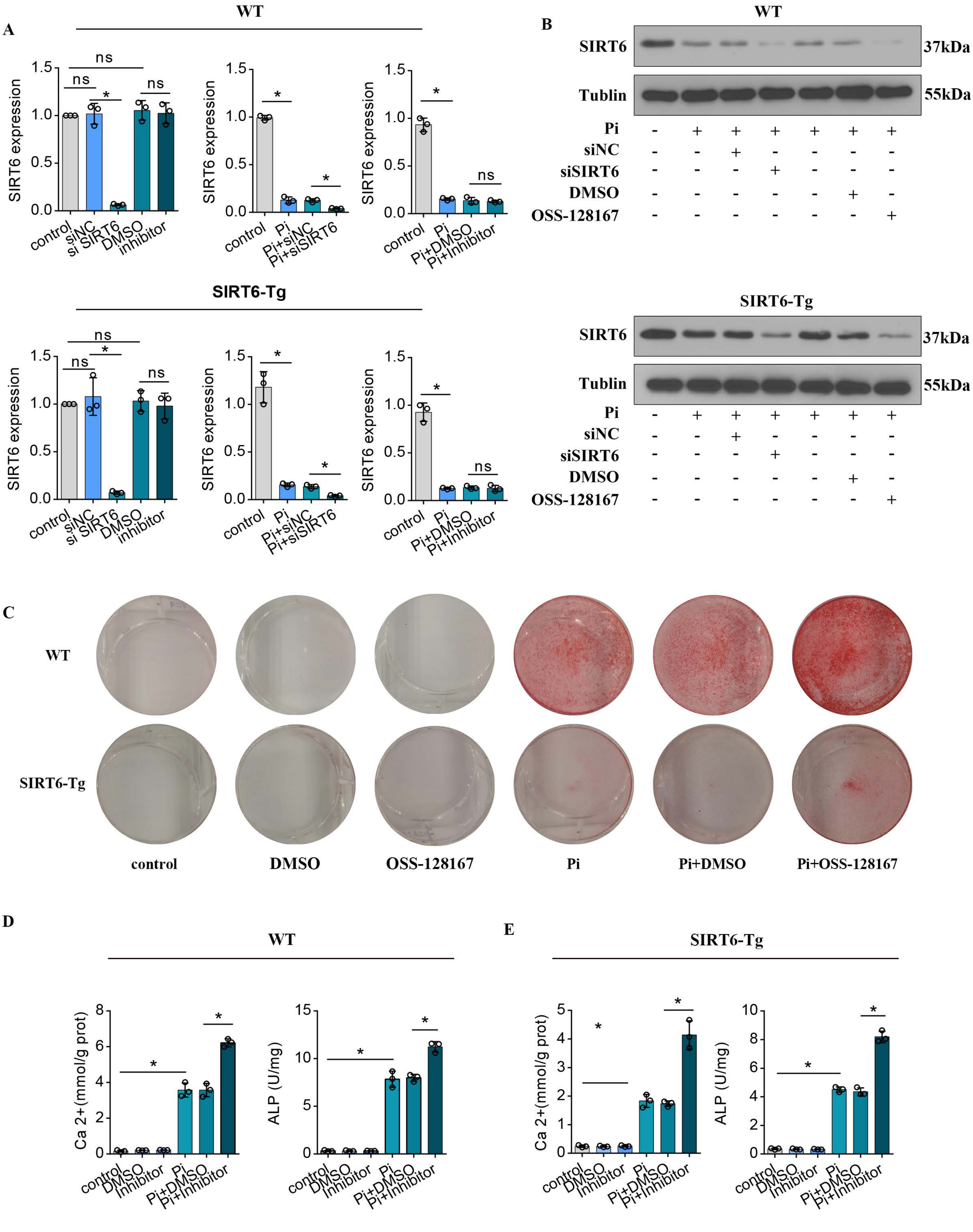
A
WT
SIRT6 expression
1.5
1.0
0.5
0.0
ns
ns
*
ns
control
siNC
si SIRT6
DMSO
inhibitor
control
Pi
Pi+siNC
Pi+siSIRT6
control
Pi
Pi+DMSO
Pi+Inhibitor
SIRT6-Tg
B
WT
SIRT6
Tublin
37kDa
55kDa
Pi
siNC
siSIRT6
DMSO
OSS-128167
SIRT6-Tg
C
WT
SIRT6-Tg
control
DMSO
OSS-128167
Pi
Pi+DMSO
Pi+OSS-128167
D
WT
Ca 2+(mmol/g prot)
ALP (U/mg)
control
DMSO
Inhibitor
Pi
Pi+DMSO
Pi+Inhibitor
E
SIRT6-Tg

Supplemental Fig. 7

A, B. WT and SIRT6-Tg VSMC were pre-transfected with siSIRT6 or si-negative control (siNC) and then exposed to Pi for 7 days; The VSMC was incubated with Pi together with DMSO or OSS-128167 for 7 days. The SIRT6 expression were analyzed by qPCR (**A**), and the cells extracts were immunoblotted for the indicated proteins (**B**). **C-E.** WT and SIRT6-Tg VSMC were incubated with Pi together with DMSO or OSS-128167 for 7 days. The VSMC was stained for mineralization by Alizarin red S (**C**), and the quantitative analysis of calcium content and ALP (**D-E**) were detected respectively. Statistical significance was assessed using one-way ANOVA followed by Dunnett's test and is presented as follows: $*P < 0.05$. All values are means ± S.D.

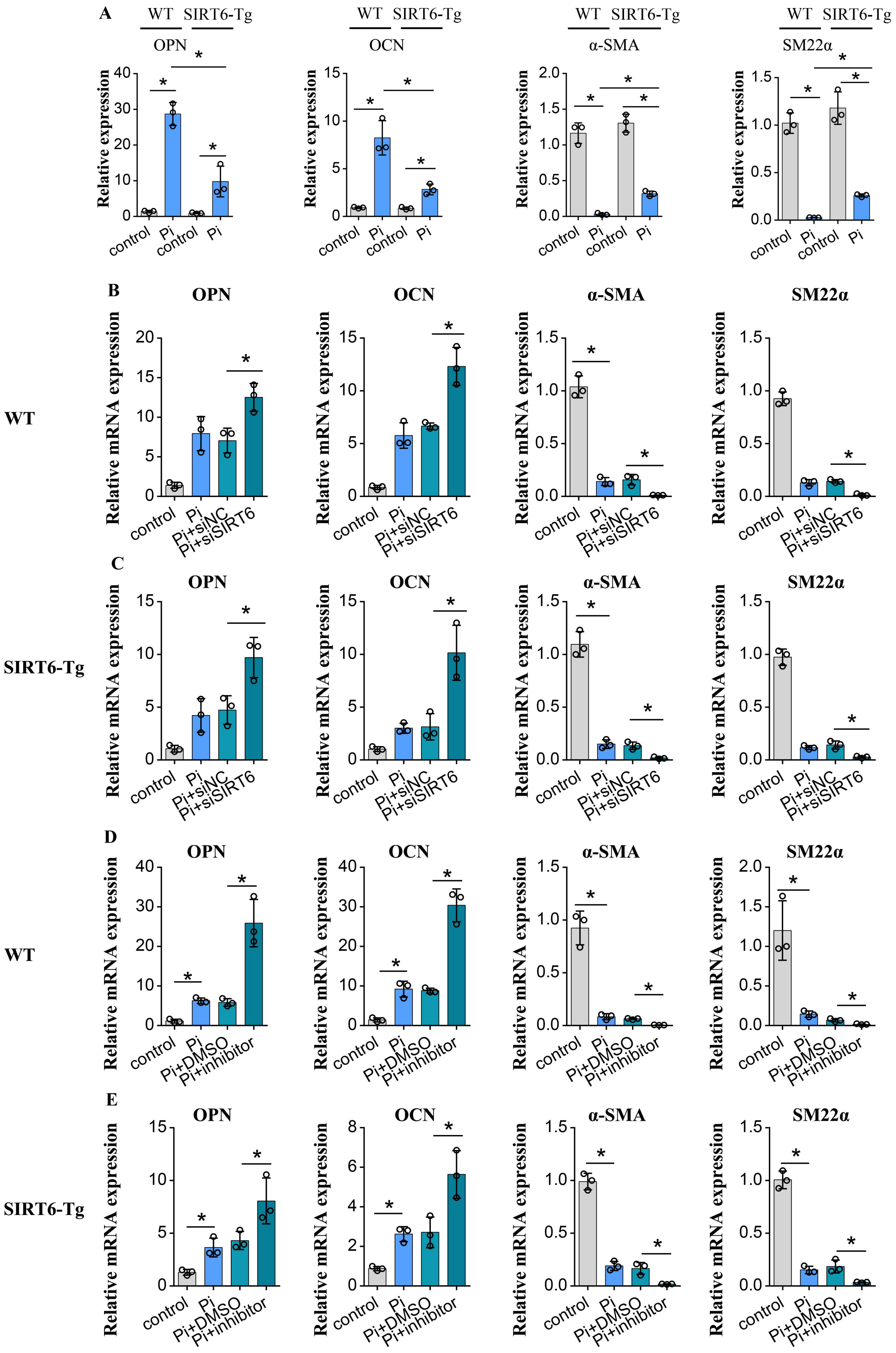
A
WT SIRT6-Tg
OPN
OCN
α-SMA
SM22α
Relative expression
control
Pi
B
C
D
E
WT
SIRT6-Tg
Relative mRNA expression
Pi+siNC
Pi+siSIRT6
Pi+DMSO
Pi+inhibitor
*

Supplemental Figure 8

A. Analysis of osteogenic and contractile property factor expression in WT and SIRT6-Tg VSMCs after Pi treatment by qPCR. **B-E.** The VSMC was pre-transfected with siSIRT6 or siNC together with Pi for 7 days, and the downstream osteogenic markers (OPN, OCN) and the contractile property marker (α-SMA, SM-22α) were analyzed by qPCR (**B, C**); VSMC was incubated with DMSO or OSS-128167 together with Pi for 7days and the same markers like above were analyzed by qPCR (**D, E**). Statistical significance was assessed using one-way ANOVA followed by Dunnett's test and is presented as follows: *$P < 0.05$. All values are means ± S.D.

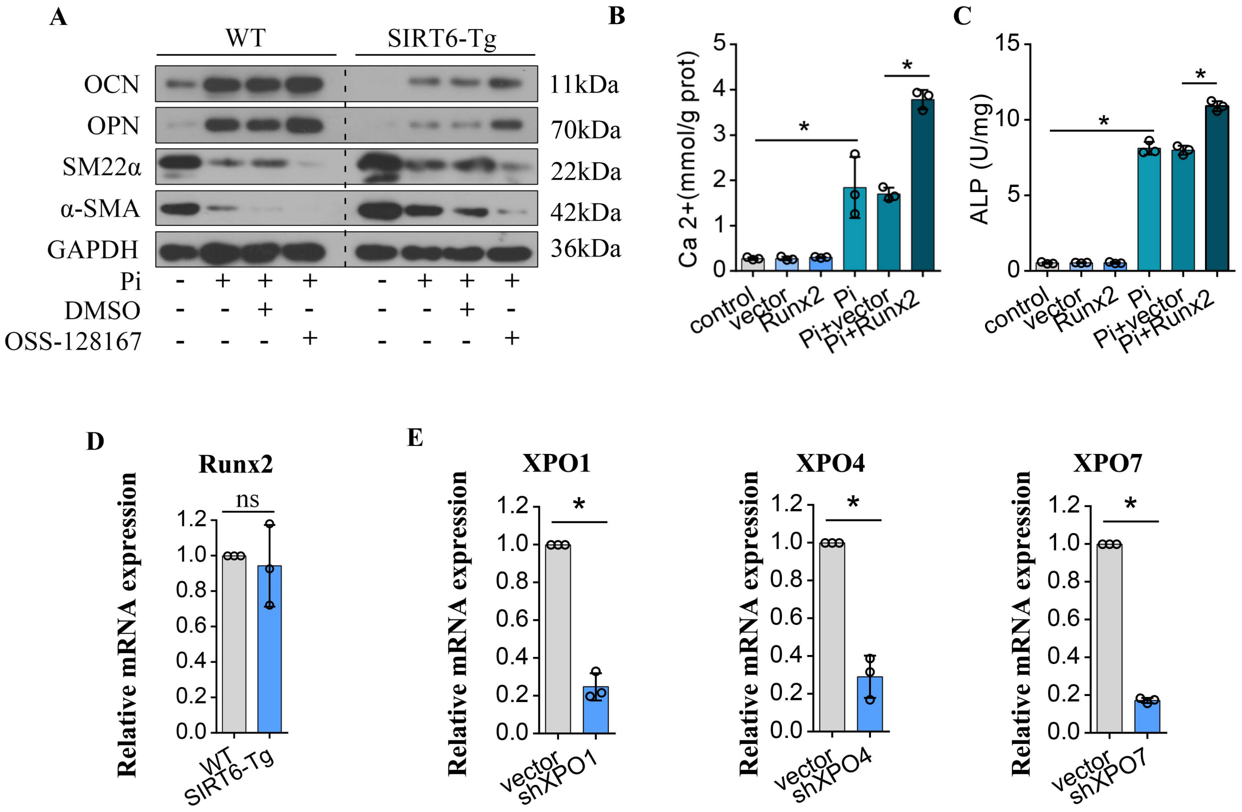

Supplemental Figure 9

A. VSMCs were incubated with DMSO or OSS-128167 (0.1mM), a SIRT6 specific inhibitor, together with Pi (3.0Mm) for 7days and the osteogenic and contractile property markers were analyzed by Western blot. **B-C.** SIRT6-Tg VSMCs were pre-transfected with Runx2 plasmid or vector plasmid, and then exposed to Pi (3.0mM) for 7 days. calcium content (**B**) and ALP (**C**) were quantified in the VSMCs. **D.** The VSMC was incubated with Pi for 7 days, then the mRNA level of Runx2 was detected through qPCR. **E.** SIRT6-Tg VSMC was transfected with shRNA targeting XPO1, XPO4, XPO7, or their vector negative control shRNA. The relative expression of XPO1, XPO4, XPO7 were analyzed by qPCR. Statistical significance was assessed using one-way ANOVA followed by Dunnett's test (**B-C**)and two-tailed t-test for two groups (**D-E**) and is presented as follows: *P < 0.05. All values are means ± S.D.

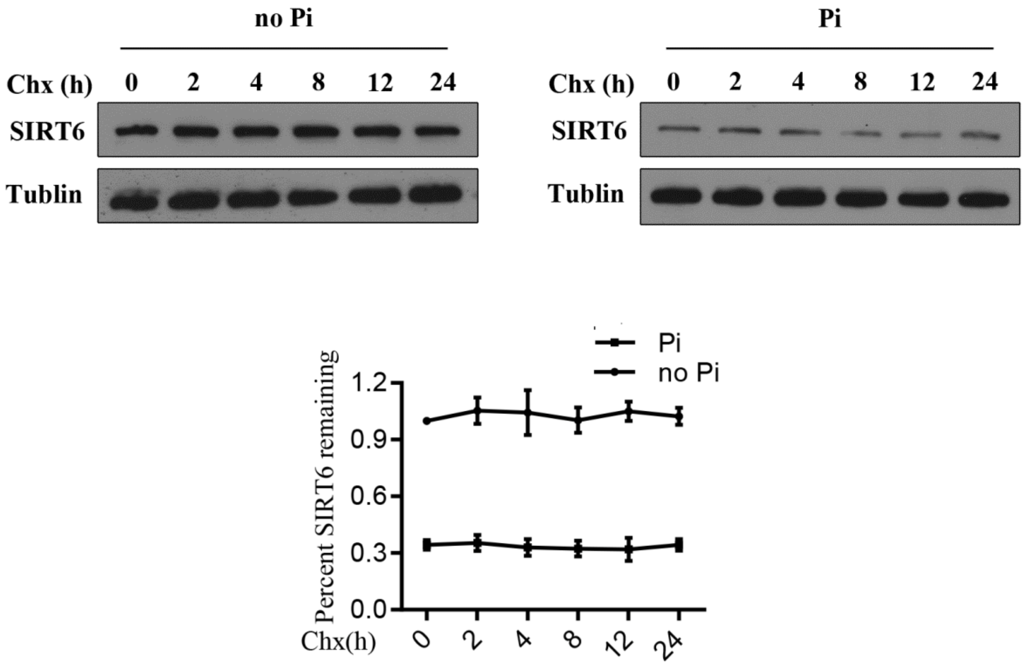

Supplemental Figure 10

WT VSMCs were treated with Pi for 7 days and incubated with the protein translation inhibitor CHX (0.2mM) for the indicated times before harvest, followed by immunoblotting with the anti-SIRT6 antibody and anti-Tublin anti-body. The curve shows the stability of SIRT6 protein (The SIRT6 protein of Pi group were normalized by no Pi group).

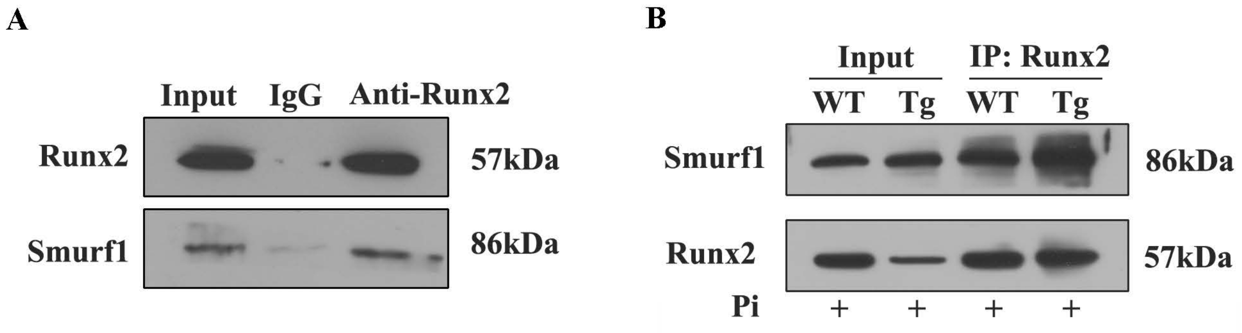

Supplemental Figure 11

A. Anti-Runx2 IP followed by WB with anti-Runx2 or anti-Smurf1 antibody in WT VSMCs after treatment with Pi for 7days. Anti-rabbit IgG IP was used as a negative control. **B**. Anti-Runx2 IP in WT and SIRT6-Tg VSMCs after treatment with Pi for 7days. WB was carried out with anti-Runx2 and anti-Smurf1 antibody. Anti-rabbit IgG IP was used as a negative control.

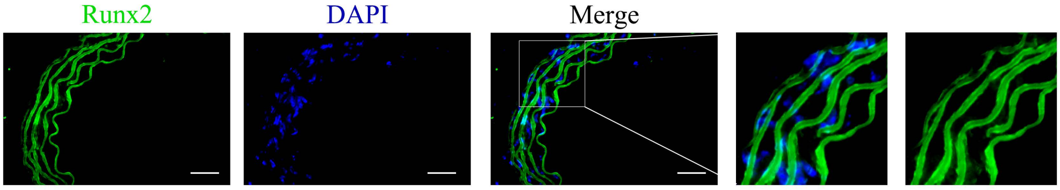

Supplemental Figure 12

The aortas slides were incubated with anti-rabbit-FITC antibody for 1h at room temperature.Nuclei were counterstained with DAPI. Prolong Gold antifade reagent was used to decrease fluorescence quenching of the slides. The elastic fibers are fluorescing. The picture shown as a negative control for IF staining. Scale bar, 50μm

Supplemental Table1: The Primers of Relative Genes in this work

Gene Name	Primers
hSIRT1 Forward(5'- 3')	GGAGCAGATTAGTAGGCGGC
hSIRT1 Reverse(5'- 3')	ACCTCAGCGCCATGGAAAAT
hSIRT2 Forward(5'- 3')	TCCTGCGGAACTTATTCTCCC
hSIRT2 Reverse(5'- 3')	GATGGTTGGCTTGAACTGCC
hSIRT3 Forward(5'- 3')	CCCAGTGGCATTCCAGACTT
hSIRT3 Reverse(5'- 3')	AAGGGCTTGGGGTTGTGAAA
hSIRT4 Forward(5'- 3')	CTCGAAAGCCTCCATTGGGT
hSIRT4 Reverse(5'- 3')	GGCCAGCCTACGAAGTTTCT
hSIRT5 Forward(5'- 3')	AGGAAAAGGGTGTGAAGAGGC
hSIRT5 Reverse(5'- 3')	GGAAGTGCCCACCACTAGAC
hSIRT6 Forward(5'- 3')	ACGCAGTACGTCAGAGACAC
hSIRT6 Reverse(5'- 3')	GTTGACAATGACCAGACGGC
mSIRT6 Forward(5'- 3')	CACAAAACATGACCGCCAGG
mSIRT6 Reverse(5'- 3')	CTGCACCATTGAGATGCACG
hSIRT7 Forward(5'- 3')	CTTGGTCGTCTACACAGGCG
hSIRT7 Reverse(5'- 3')	GGTGATGCTCATGTGGGTGA
mRunx2 Forward(5'- 3')	ATCCCCATCCATCCACTCCA
mRunx2 Reverse(5'- 3')	GGGGTGTAGGTAAAGGTGGC
mSM22αForward(5'- 3')	GGTCCATCCTACGGCATGAG
mSM22αReverse(5'- 3')	TGCTCCTGGGCTTTCTTCATA
mα-SMA Forward(5'- 3')	GTACCACCATGTACCCAGGC
mα-SMA Reverse(5'- 3')	GCTGGAAGGTAGACAGCGAA
mOCN Forward(5'- 3')	GGTAGTGAACAGACTCCGGC
mOCN Reverse(5'- 3')	TTAAGCTCACACTGCTCCCG
mOPN Forward(5'- 3')	CCTGGCTGAATTCTGAGGGAC
mOPN Reverse(5'- 3')	CAGTCACTTTCACCGGGAGG
mXPO1 Forward(5'- 3')	TGAACACGAAATACTATGGACTACA
mXPO1 Reverse(5'- 3')	CTGGTCCTACTCGCTCCAAC
mXPO4 Forward(5'- 3')	AAAAGGGCAGCATCGAGTCA
mXPO4 Reverse(5'- 3')	TGCCACTACTTATCAACTGGCT
mXPO7 Forward(5'- 3')	CAAAGCTGGGCTGGTTTGAC
mXPO7 Reverse(5'- 3')	AGTTGTGTCTGCTTGATTAATTTCA

Supplementary Table 2 : The Relative siRNA and shRNA in this work

Gene name	Relative sequences (5’ - 3’)
SIRT6 siRNA	CATGTTTCGTATAAGTCCAA
XPO1 shRNA	CCGGGATTATGTAGATACGGAAATACTCGAGTATTTCCG TATCTACATAATCTTTTTTG
XPO4 shRNA	CCGGGCTACCTCTTAGCTGATGATACTCGAGTATCATCAG CTAAGAGGTAGCTTTTTG
XPO7 shRNA	CCGGCCCTGATGTTATCCGATTGATCTCGAGATCAATCGG ATAACATCAGGGTTTTTG

Supplementary Table 3 :The antibodies used in this study

Anti-SIRT6	Abcam #ab191385	WB 1:1000; IHC/IF 1:200; IP: 5μl for 1mg protein
Anti-Runx2	Abcam #ab76956	WB 1:1000; IF 1:200
Anti-Runx2	CST #12556s	IP: 5μl for 1mg protein
Anti-α-SMA	Abclonal #A17910	WB 1:1000; IF 1:200
Anti-SM22α	Proteintech #10493-1-AP	WB 1:1000; IF 1:200
Anti-OCN	Proteintech #23418-1-AP	WB 1:1000; IF 1:200
Anti-OPN	Proteintech #22952-1-AP	WB 1:1000; IF 1:200
Anti-XPO1	Abcam #ab180144	WB 1:1000; IP: 5μl for 1mg protein
Anti-HA	CST #3724S	WB 1:1000; IP: 5μl for 1mg protein
Anti-Flag	CST #14793S	WB 1:1000; IP: 5μl for 1mg protein
Anti-Ub	CST #43124S	WB 1:1000
Anti-Acetylased-lysine	CST #9441S	WB 1:1000
Anti-Smurf1	Affinity #DF7713	WB 1:1000
Anti-Histone3	Abclonal #A2352	WB 1:1000
Anti-GAPDH	Proteintech # 60004-1-Ig	WB 1:1000
Anti-tublin	Proteintech #11224-1-AP	WB 1:1000
Anti-β-actin	Proteintech #66009-1-Ig	WB 1:1000
Anti-rabbit- FITC	Sigma # F9887	IF: 1:2000
Anti-mouse-Alexa Flu-647	Abcam # ab150115	IF: 1:2000
Anti-rabbit	CWBIO # CW0103	WB: 1:10000
Anti-mouse	CWBIO # CW0102	WB: 1:10000
IgG	CST #2729	IP: 2μl for 1mg protein
